# Impact of Regulation of Wax-Based and Bio-Oil-Based Warm-Mix Additives on the Phase Behavior and Rheological Properties of Rubber-Modified Asphalt

**DOI:** 10.3390/ma19173613

**Published:** 2026-08-25

**Authors:** Wenqi Wang, Jiawei Huang, Hongyu Bai, Hongxi Luo, Weian Xuan, Mingming Cao

**Affiliations:** 1 School of Architecture and Civil Engineering, Xihua University, Chengdu 610039, China; wangwenqi@xhu.edu.cn (W.W.);; 2 School of Civil Engineering, Southwest Jiaotong University, Chengdu 610031, China; 3 Guangxi Key Laboratory of Road Structure and Materials, Guangxi Transportation Science and Technology Group Co., Ltd., Nanning 530029, China; 4 Sichuan Communication Surveying & Design Institute Co., Ltd., Chengdu 610041, China

**Keywords:** rubber-modified asphalt, warm-mix additive, phase behavior, rheological properties, performance-related indicators

## Abstract

Two warm-mix modification routes were examined to determine how additive chemistry affects the service-temperature rheology of rubber-modified asphalt. Wax-based and bio-oil-based additives were incorporated at 1–3%, and the resulting binders were characterized by FTIR, dynamic shear rheology, MSCR, LAS, and BBR testing. These measurements respectively provided physicochemical evidence and quantified the phase-related response, deformation recovery, fatigue-related damage tolerance, and low-temperature relaxation. The wax-based system developed a stiffness-oriented response: an intermediate dosage produced comparatively lower *J*_nr_ and higher *R*, but further addition impaired relaxation, with *m*(60) decreasing to 0.285 at −18 °C for the 3% formulation. In contrast, the bio-oil-based system favored relaxation; at a 3% dosage, the LAS-predicted *N_f_* at 2% strain was 15,200 cycles, while *m*(60) reached 0.460 at −12 °C and 0.384 at −18 °C. The binder-level evidence therefore identifies different selection priorities: an intermediate wax dosage is advantageous when deformation recovery is emphasized, whereas the bio-oil-based route is more favorable for relaxation and low-temperature response. Additional mixture and workability testing is required before these binder findings are translated into construction-temperature or field-performance recommendations.

## 1. Introduction

Recycling waste-tire rubber in asphalt binders can increase the elastic response and resistance to permanent deformation while providing a productive outlet for discarded tires [1,2,3,4]. This benefit is accompanied by a substantial viscosity increase, which complicates mixing, placement, and compaction, especially when lower production temperatures are desired [5,6,7]. Warm-mix technologies have consequently been introduced to reduce the thermal demand of rubberized binders and facilitate construction [8,9,10,11,12]. Although their workability benefits are well documented, the present work addresses a different question: how these additives modify phase-related rheological behavior under service-temperature conditions [8,9,10,11,12].

Viscosity reduction alone does not describe the material changes produced by warm-mix additives. Crumb-rubber particles take up lighter asphalt fractions during swelling and develop an interfacial region whose composition influences the overall viscoelastic response [13,14,15]. An additive may alter this state through softening, crystallization, physical structuring, or interactions involving functional groups [16,17,18]. The resulting balance among stiffness, recovery, and stress relaxation can therefore differ even when two products provide comparable warm-mix effects. A mechanistic evaluation must consequently consider several rheological indicators rather than infer performance from viscosity or any single parameter.

Published investigations have generally assessed warm-mix rubberized binders through viscosity, high-temperature stability, fatigue-related response, or low-temperature cracking indicators [10,12,17]. These individual tests are valuable for engineering screening, yet they do not fully explain how additive-induced changes in the internal phase state lead to the observed rheological response. In a rubber–asphalt system, phase behavior encompasses compatibility, relaxation-spectrum characteristics, and the coupling of viscous and elastic contributions [19,20,21]. Treating this behavior as the connection between additive action and performance-related binder indicators offers a more integrated basis for interpreting formulation effects.

The experimental sequence was designed to connect physicochemical observations with rheological evidence. Fourier transform infrared spectroscopy (FTIR) first identified changes in characteristic absorption bands associated with crumb rubber and the two additives [18,22]. Strain-amplitude response then described network stability and large-deformation rearrangement [23], while frequency-dependent analyses compared broadband viscoelasticity, thermorheological behavior, and relaxation-time distribution through master curves, Black diagrams, and Cole–Cole plots [24,25,26,27,28,29]. Finally, multiple stress creep recovery (MSCR), linear amplitude sweep (LAS), and bending beam rheometer (BBR) testing supplied binder-scale indicators related to deformation recovery, fatigue-damage tolerance, stiffness, and stress relaxation [30,31,32]. Together, these measurements provide complementary evidence for the binder contribution to rutting, fatigue cracking, and thermal cracking without treating any single indicator as a direct measure of mixture or pavement performance.

The study therefore compares wax-based and bio-oil-based warm-mix formulations across a controlled dosage range. Its objective is to establish how the additive type and concentration affect stiffness, relaxation, deformation recovery, and fatigue-related damage indicators in rubber-modified asphalt. The resulting binder-level relationships are intended to support additive selection and to define hypotheses for subsequent mixture-performance, workability, and field validation.

## 2. Materials and Methods

### 2.1. Materials and Sample Preparation

#### 2.1.1. Raw Materials

Eight binder formulations were included in the experimental program. The constituents were 70# base asphalt (Kunlun brand, PetroChina Fuel Oil Co., Ltd., Zhuhai, China), 80-mesh crumb rubber produced from waste tires (Hebei Kexu Building Materials Co., Ltd., Shijiazhuang, China), and commercial wax-based and bio-oil-based warm-mix additives. Unmodified base asphalt was designated QP, and rubber-modified asphalt without either warm-mix product served as the CRMA control. Each additive was introduced into CRMA at 1%, 2%, or 3% of total binder mass, producing the sample designations summarized in Table 1.

Table 2 summarizes the conventional physical properties of the base asphalt, whereas Table 3 reports the measured composition-related properties of the crumb-rubber powder.

Both additives used in this study are supplied by their respective manufacturers as commercial warm-mix additives specifically developed for asphalt applications, and their main physicochemical properties are described as follows. The wax-based additive is Sasobit^®^, a Fischer-Tropsch synthetic wax supplied by Sasol (Johannesburg, South Africa). At ambient temperature, it appears as white spherical solid pellets and consists mainly of long-chain saturated straight-chain alkanes ranging from *C*_40_ to *C*_120_, with a melting range of 100–110 °C, a crystallization range of 95–105 °C, and a penetration at 25 °C of 0–20 (0.1 mm). The bio-oil-based additive is a commercial modified vegetable-oil derivative obtained from a commercial supplier (Chengdu, China), prepared by esterification of agricultural and forestry vegetable oils. It appears as a brown liquid at ambient temperature and consists mainly of unsaturated fatty acid methyl esters and glycerol ester derivatives, with a density of 0.92 g/cm^3^ and a viscosity of 0.175 Pa·s at 60 °C.

#### 2.1.2. Sample Preparation

**Figure 1:**
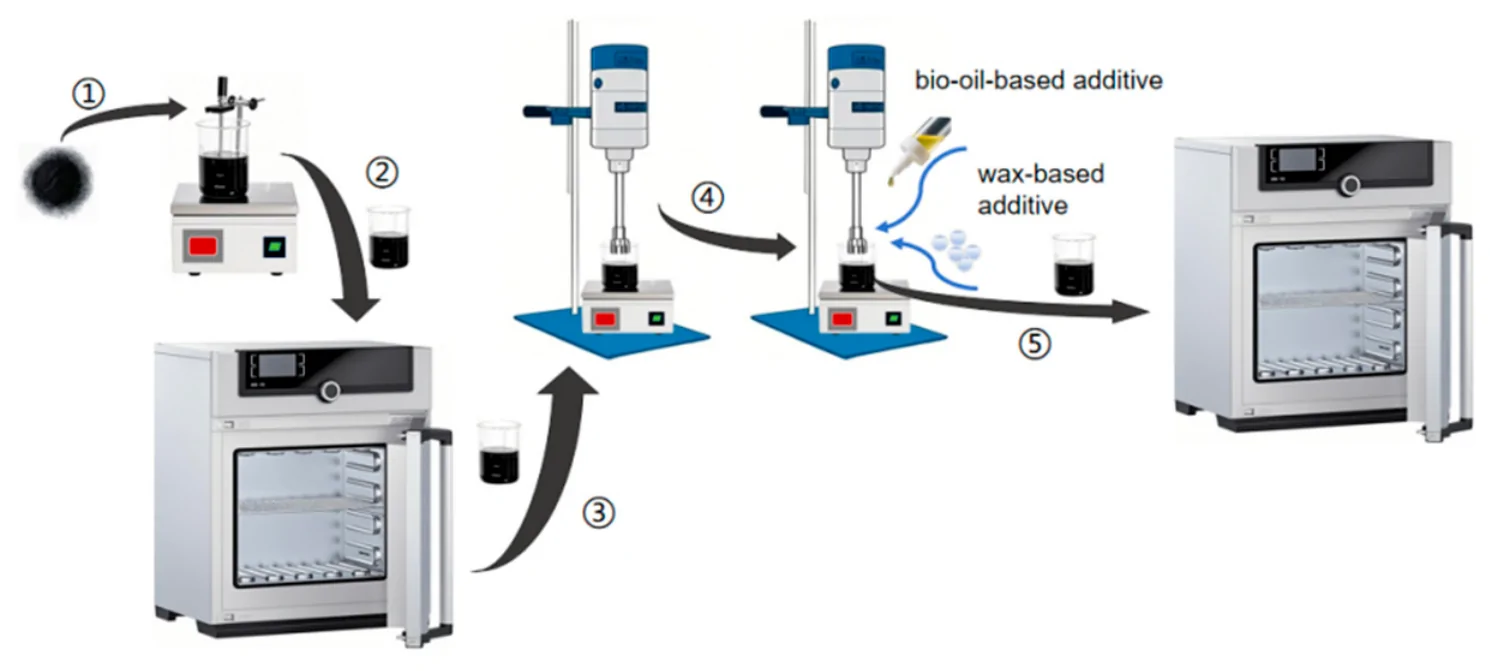
Preparation procedure of asphalt binders.

The wet-process sequence used to produce CRMA is illustrated in Figure 1. Base asphalt was first brought to 170 °C, after which crumb rubber equivalent to 8% of the total binder mass was introduced and mechanically stirred at 4500 r/min for 1 h to obtain a macroscopically uniform initial dispersion. The mixture was then placed in a forced-air oven at 185 °C for 30 min without agitation. This quiescent conditioning stage maintained a stable thermal environment and allowed the rubber particles to absorb lighter binder fractions and swell without continuous shear disturbing the developing asphalt–rubber interfacial structure. It was subsequently processed for 60 min at 180 °C and 4500 r/min using a high-shear mixer to obtain CRMA [33].

Each warm-mix formulation was produced from the prepared CRMA by introducing the selected additive at 1%, 2%, or 3% and continuing high-shear mixing for a further 30 min at 180 °C. All resulting binders were subsequently conditioned at 185 °C for 120 min so that the modified samples experienced the same post-preparation thermal history. The complete set comprised QP, CRMA, three wax-modified binders (CRMAL1–CRMAL3), and three bio-oil-modified binders (CRMAZ1–CRMAZ3).

It should be noted that both the preparation procedure and the selection of the crumb rubber content were based on our previous work [33], which demonstrated that this method yields stable swelling of rubber particles and a fully developed asphalt–rubber interaction. The 1%, 2%, and 3% additive dosages were adopted as a controlled-variable range covering low, medium, and high dosages, so that the direction and dose dependence of the rheological and phase-behavior effects of each additive could be identified under an otherwise identical binder system.

All rubber-modified binders (CRMA, CRMAL, and CRMAZ series) received an identical thermal history throughout preparation, so that observed differences among these samples can be attributed to the additive type and dosage rather than to differences in preparation history. The base asphalt QP was not subjected to the wet-process procedure and is used solely as the reference for neat-binder properties.

Prolonged high-temperature conditioning at 185 °C may induce a limited degree of short-term binder aging, additional rubber digestion, or partial volatilization of light components of the bio-oil-based additive. All rubber-modified binders received identical thermal exposure; therefore, these effects are systematic across the samples, and the relative comparisons among additive types and dosages remain valid.

### 2.2. Experimental Characterization

#### 2.2.1. FTIR Test

Attenuated total reflectance Fourier transform infrared (ATR-FTIR) spectra were acquired with a Nicolet™ iS20 instrument (Thermo Fisher Scientific, Waltham, MA, USA) operated in absorbance mode. Each binder was warmed only enough to permit homogenization, transferred onto the cleaned ATR crystal, and allowed to return to room temperature while maintaining uniform crystal contact. A fresh background was recorded before every specimen, and the crystal was cleaned between measurements. Data were collected from 4000 to 400 cm^−1^ with a spectral resolution of 0.25 cm^−1^ and 32 co-added scans. At least three independently selected positions were measured for each binder, and their average spectrum was used for qualitative interpretation. The analysis focused on the principal absorption bands as initial physicochemical evidence, which was subsequently considered alongside the rheological results.

#### 2.2.2. Dynamic Shear Rheological Characterization

**Figure 2:**
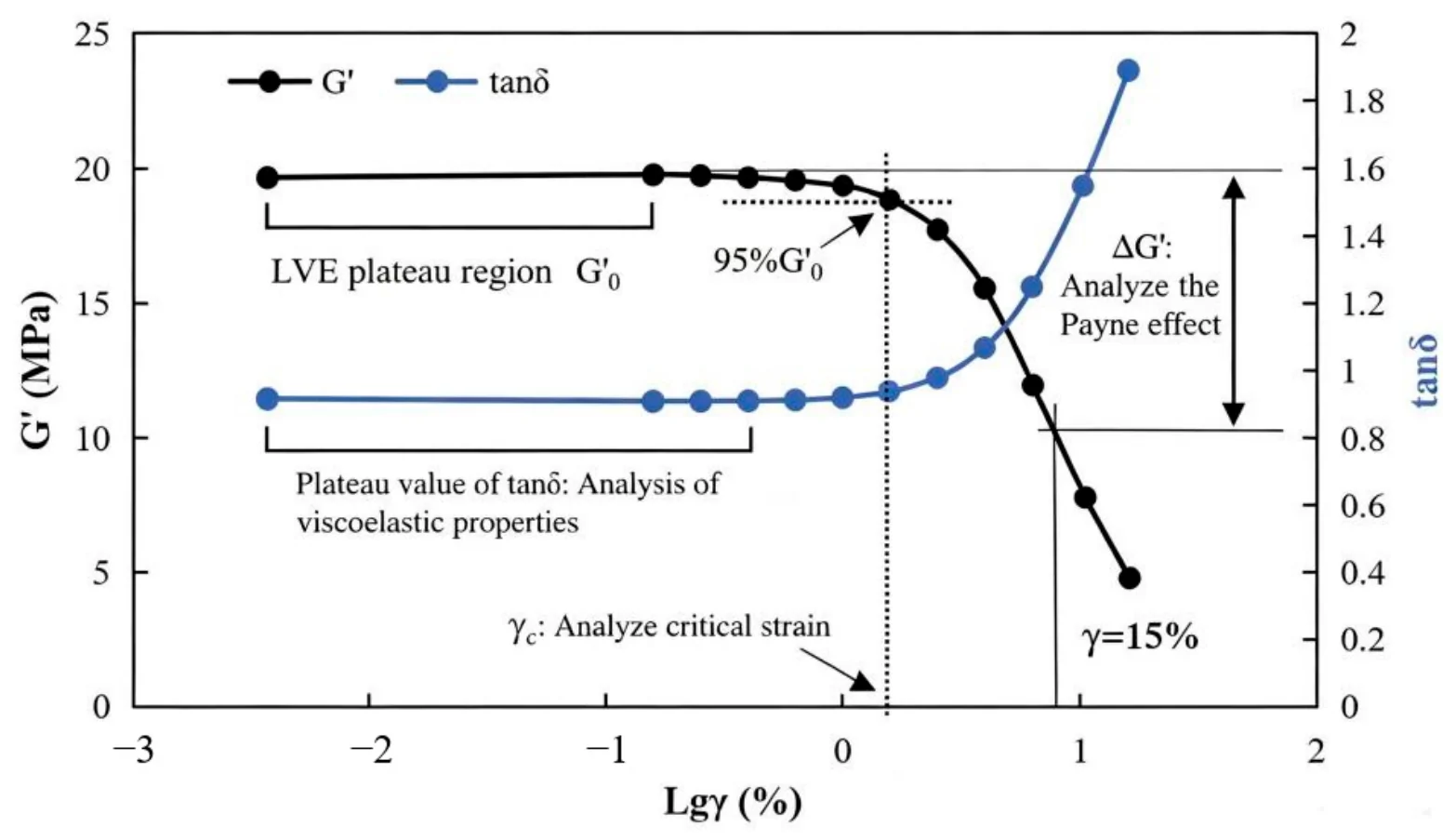
Schematic strain sweep curves and definitions of key parameters.

Strain-amplitude measurements were performed with a Discovery HR-3 dynamic shear rheometer (TA Instruments, New Castle, DE, USA) to establish the linear viscoelastic (LVE) range and characterize a nonlinear network response. Tests were run in strain control at 5, 20, and 35 °C over 0.1–20% strain while the angular frequency was maintained at 10 rad/s. For *G*′ and tan*δ*, the plateau was calculated as the mean of the stable data points preceding the nonlinear response. The critical strain *γ*_c_ corresponded to a 5% decrease in *G*′ from its plateau; this conventional threshold was used to define the LVE boundary [34]. The loss factor tan*δ* was calculated as *G*″/*G*′. Large-strain modulus reduction was retained as the absolute quantity Δ*G*′ = *G*′_0_ − *G*′_15%_. The 15% strain point was selected because every binder had entered the nonlinear regime and showed an evident modulus decrease, enabling a consistent comparison of network rearrangement under large deformation [24]. Figure 2 illustrates these definitions.

Frequency-dependent response was measured at between 0.1 and 50 rad/s at 5, 30, and 55 °C. A 25 mm parallel plate with a 1 mm gap was used at 30 and 55 °C, whereas measurements at 5 °C employed an 8 mm plate and a 2 mm gap. The applied strain for every sweep remained within the previously determined LVE range. Data from the three temperatures were shifted horizontally to a reference temperature of 30 °C to generate G′ and G″ master curves [26]. Williams–Landel–Ferry (WLF) coefficients were not estimated because three temperature levels are insufficient for reliable parameter fitting; the experimentally selected shift factors were used directly. Black and Cole–Cole representations were then derived from the same frequency-sweep data to compare phase continuity, relaxation distribution, and viscoelastic compatibility [27,28]. The strain- and frequency-sweep tests were conducted on unaged binders. Three independently prepared binder batches were examined for each binder in both programs, and subsequent analyses used the corresponding averages.

#### 2.2.3. High-Temperature Deformation and Recovery (MSCR)

The multiple stress creep recovery (MSCR) test was employed to evaluate the high-temperature rutting resistance of binders, following AASHTO T 350. Prior to MSCR testing, all asphalt binders were subjected to short-term aging via the Rolling Thin-Film Oven Test (RTFOT, AASHTO T 240), consistent with the mandatory binder conditioning specified in AASHTO T 350 for rutting performance evaluation. Tests were conducted at 58 °C by sequentially applying 20 creep-recovery cycles at 0.1 kPa followed by 10 cycles at 3.2 kPa. Each cycle consisted of 1 s creep loading and 9 s recovery. Non-recoverable creep compliance *J*_nr_ and the recovery percentage *R* were employed to characterize permanent-deformation resistance and elastic recovery capacity, and the stress sensitivity indices *J*_nr,diff_ and *R*_diff_ were further calculated to evaluate performance stability under different stress levels.

(1) $$J_{nr}=\frac{\varepsilon_{u}}{\sigma }$$

(2) $$R=\frac{\varepsilon_{p}-\varepsilon_{u}}{\varepsilon_{p}}\times 100\%$$

(3) $$J_{nr,diff}=\frac{J_{nr,3.2}-J_{nr,0.1}}{J_{nr,0.1}}\times 100\%$$

(4) $$R_{diff}=\frac{R_{0.1}-R_{3.2}}{R_{0.1}}\times 100\%$$

where *J*_nr_ is the non-recoverable creep compliance, *ε_u_* is the non-recoverable strain after the recovery stage, *σ* is the applied shear stress, *R* is the recovery percentage, and *ε_p_* is the peak strain during the creep-loading stage. For each specimen, *J_nr_* and *R* were calculated for each creep-recovery cycle using Equations (1) and (2). At 0.1 kPa, 20 cycles were applied, and the averages of cycles 11–20 were reported; at 3.2 kPa, all 10 applied cycles were averaged, in accordance with AASHTO T 350. The final reported value for each binder was the mean obtained from three independently prepared binder batches.

#### 2.2.4. Intermediate-Temperature Fatigue-Related Response (LAS)

RTFOT-aged binders were evaluated by LAS at 25 °C in accordance with AASHTO T 391; the preceding RTFOT conditioning followed AASHTO T 240 and represented short-term aging associated with mixing and placement. An 8 mm parallel plate and a 2 mm gap were used. The first stage was a frequency sweep from 0.2 to 30 Hz at 0.1% strain to establish undamaged viscoelastic properties. This was followed by a 300 s amplitude sweep at 10 Hz in which the strain increased linearly from 0.1% to 30%. The progressive modulus decrease during this stage supplied the damage-evolution data used in the subsequent VECD analysis.

The fatigue analysis was performed using viscoelastic continuum damage (VECD) theory, which is grounded in Schapery’s elastic–viscoelastic correspondence principle and continuum damage mechanics. This framework decouples the intrinsic viscoelastic response of the material from damage-induced stiffness reduction by correlating the modulus degradation rate with the strain history and the material’s own damage characteristics. The damage parameter *D*(*t*) was calculated incrementally from the amplitude-sweep data as

(5) $$D(t)≅\sum_{i=1}^{N}{\left[\pi I_{D}{\gamma_{0}}^{2}({|G^{*}|sin\delta }_{i-1}-{|G^{*}|sin\delta }_{i})\right]}^{\frac{\alpha }{1+\alpha }}{\left(t_{i}-t_{i-1}\right)}^{\frac{1}{1+\alpha }}$$

where *I_D_* is the initial undamaged value of |G^*^|sin*δ* measured at the beginning of the amplitude sweep; *γ*_0_ is the applied strain amplitude; |G^*^| is the complex shear modulus (MPa); *δ* is the phase angle; *t* is the test time (s); and *α* is the damage-evolution exponent obtained from the frequency sweep as

(6) $$logG^{\prime }(\omega )=m(log\omega )+b$$

(7) $$\alpha =\frac{1}{m}$$

where *G*′(*ω*) is the storage modulus (MPa); *ω* is the angular frequency (rad/s); *m* is the fitted slope; and *b* is the regression intercept.

Based on VECD theory, the material-integrity parameter *C*(*t*) was defined by normalizing |G^*^|sin*δ* to its initial undamaged value and was related to accumulated damage as

(8) $$C(t)=\frac{{\left|G^{*}\right|}_{t}sin\delta_{t}}{I_{D}}=C_{0}-C_{1}{\left[D(t)\right]}^{C_{2}}$$

where *C*_0_ = 1, and *C*_1_ and *C*_2_ are coefficients obtained by least-squares fitting of the *C*–*D* relationship. Within the VECD framework, this fitted relationship describes the evolution of material integrity with accumulated damage under the prescribed LAS loading history.

The fatigue failure criterion was defined as the damage value *D_f_* corresponding to a 35% reduction in the initial |G^*^|sin*δ* value:

(9) $$D_{f}={\left[\frac{0.35C_{0}}{C_{1}}\right]}^{\frac{1}{C_{2}}}$$

Fatigue life *N_f_* at a prescribed strain amplitude *γ_max_* was then calculated as

(10) $$N_{f}=A_{35}{\left(\gamma_{max}\right)}^{-B}$$

(11) $$A_{35}=\frac{f{\left(D_{f}\right)}^{k}}{k{\left(\pi I_{D}C_{1}C_{2}\right)}^{\alpha }}$$

where *f* is the loading frequency (10 Hz), *k* = 1 + (1 − *C*_2_)*α*, *B* = 2*α*, and *γ_max_* is the prescribed strain amplitude (%). A higher *N_f_* indicates a longer predicted fatigue life at the same strain amplitude. All calculations were performed using Microsoft Excel 2019 (Microsoft Corporation, Redmond, WA, USA). Three independently prepared binder batches were tested for each binder, and the reported *N_f_* values are the means of the three batches.

#### 2.2.5. Low-Temperature Stiffness and Relaxation (BBR)

**Figure 3:**
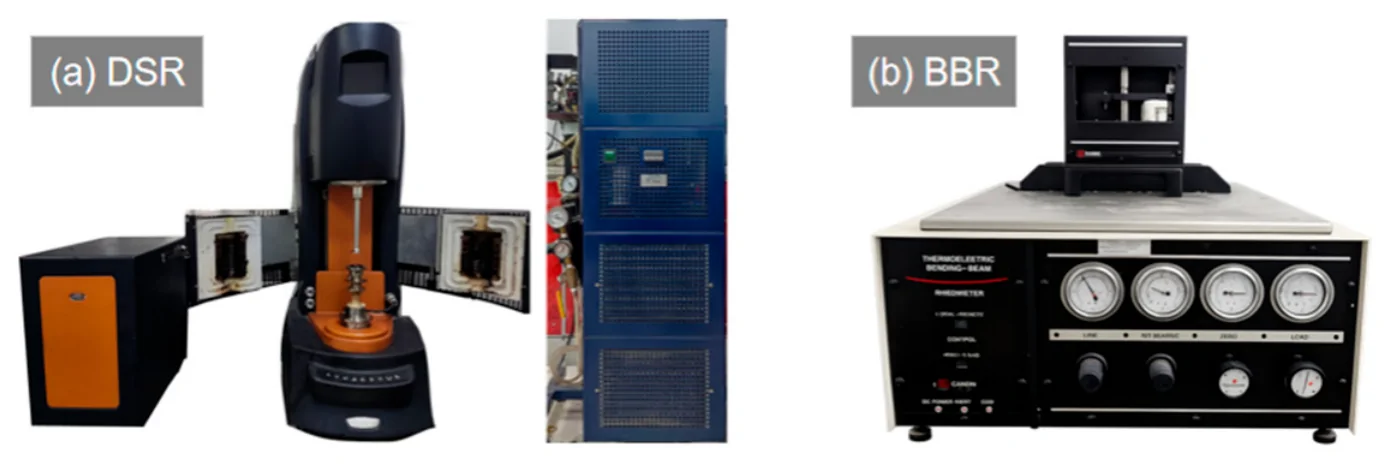
Photographs of the DSR and BBR devices.

Low-temperature creep behavior was characterized using a TE-BBR thermoelectric bending beam rheometer (Cannon Instrument Company, State College, PA, USA) in accordance with AASHTO T 313. Before beam preparation, each binder underwent RTFOT aging under AASHTO T 240 followed by pressurized aging vessel (PAV) aging under AASHTO R 28. All remaining specimen-preparation, demolding, and conditioning procedures were performed in accordance with AASHTO T 313. Tests were conducted at −12 and −18 °C after specimen conditioning at the target temperature. The creep stiffness S(60) and creep rate m(60), both evaluated at 60 s, were used to describe resistance to deformation and the capacity for stress relaxation, respectively; favorable relaxation is associated with a lower S(60) and higher m(60). Three independently prepared binder batches were evaluated for every binder–temperature combination; one beam was prepared from each batch, and the reported values are the means of the three independent batches. The DSR and BBR instruments are shown in Figure 3.

#### 2.2.6. Statistical Analysis

Statistical analysis was performed in Microsoft Excel 2019 for the LAS and BBR results using the three independently prepared binder batches for each binder and test condition. Results are reported as the mean ± standard deviation (SD). For each LAS strain level and each BBR indicator at each temperature, differences among binders were evaluated by one-way analysis of variance (ANOVA), followed by Tukey’s honestly significant difference test for pairwise multiple comparisons. Statistical significance was defined at *p* < 0.05. Different lowercase letters above the bars denote significant differences among binders within the same test condition. Groups sharing at least one lowercase letter were not significantly different, whereas groups with no common letter differed significantly according to Tukey’s test (*p* < 0.05).

## 3. Results

### 3.1. FTIR Physicochemical Characterization

**Figure 4:**
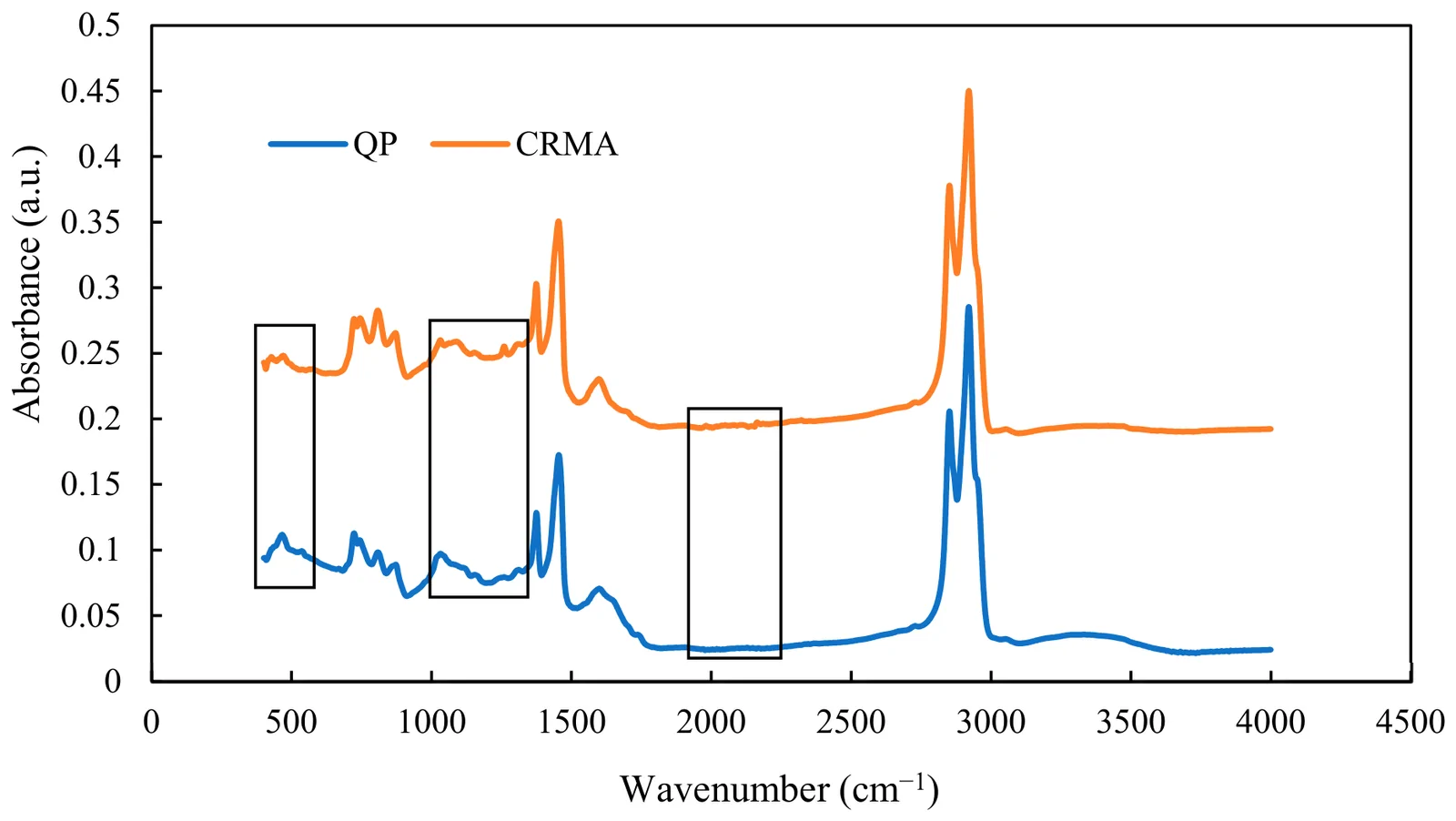
FTIR spectra of QP and CRMA samples.

**Figure 5:**
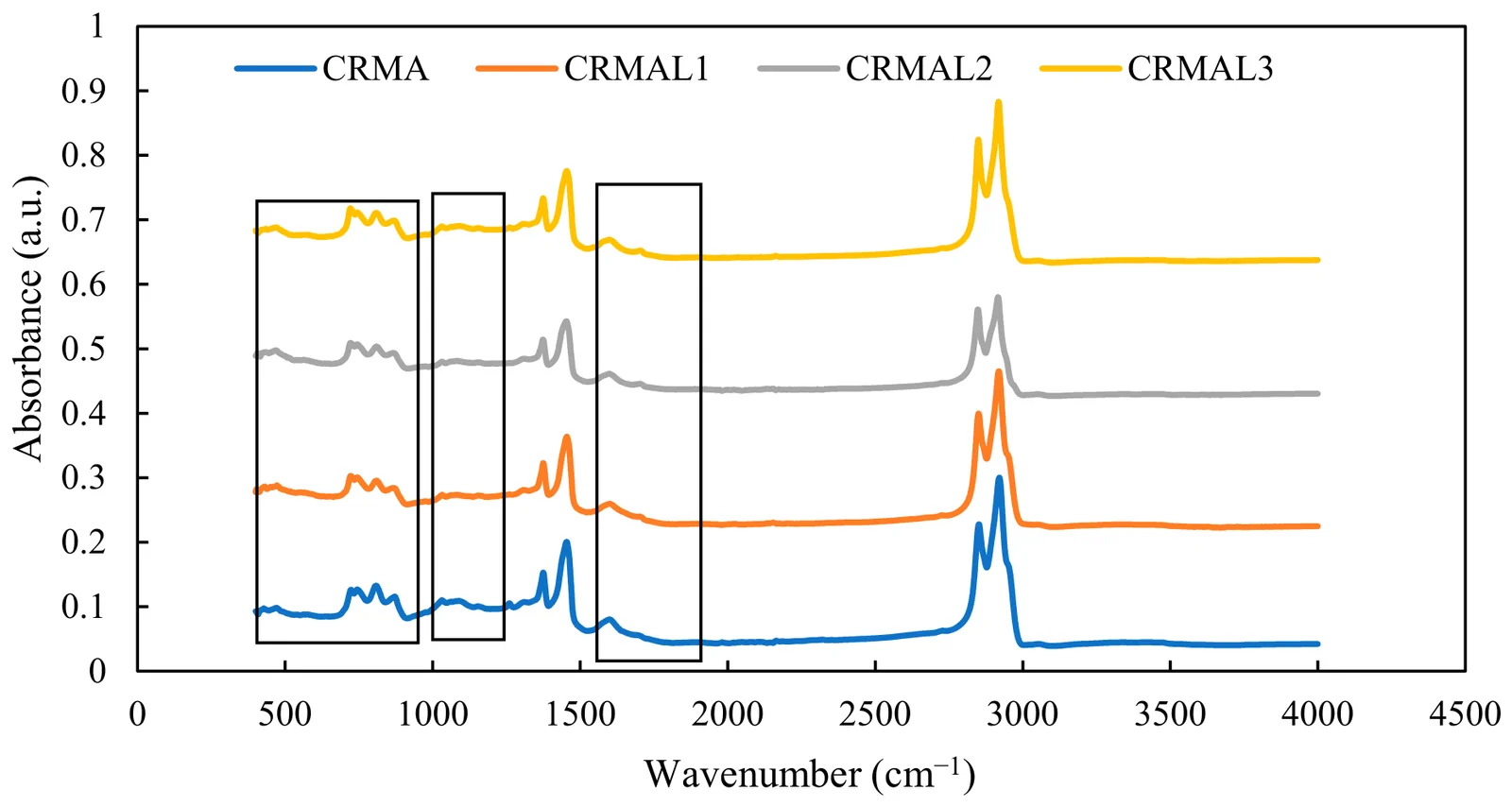
FTIR spectra of CRMAL-series samples.

**Figure 6:**
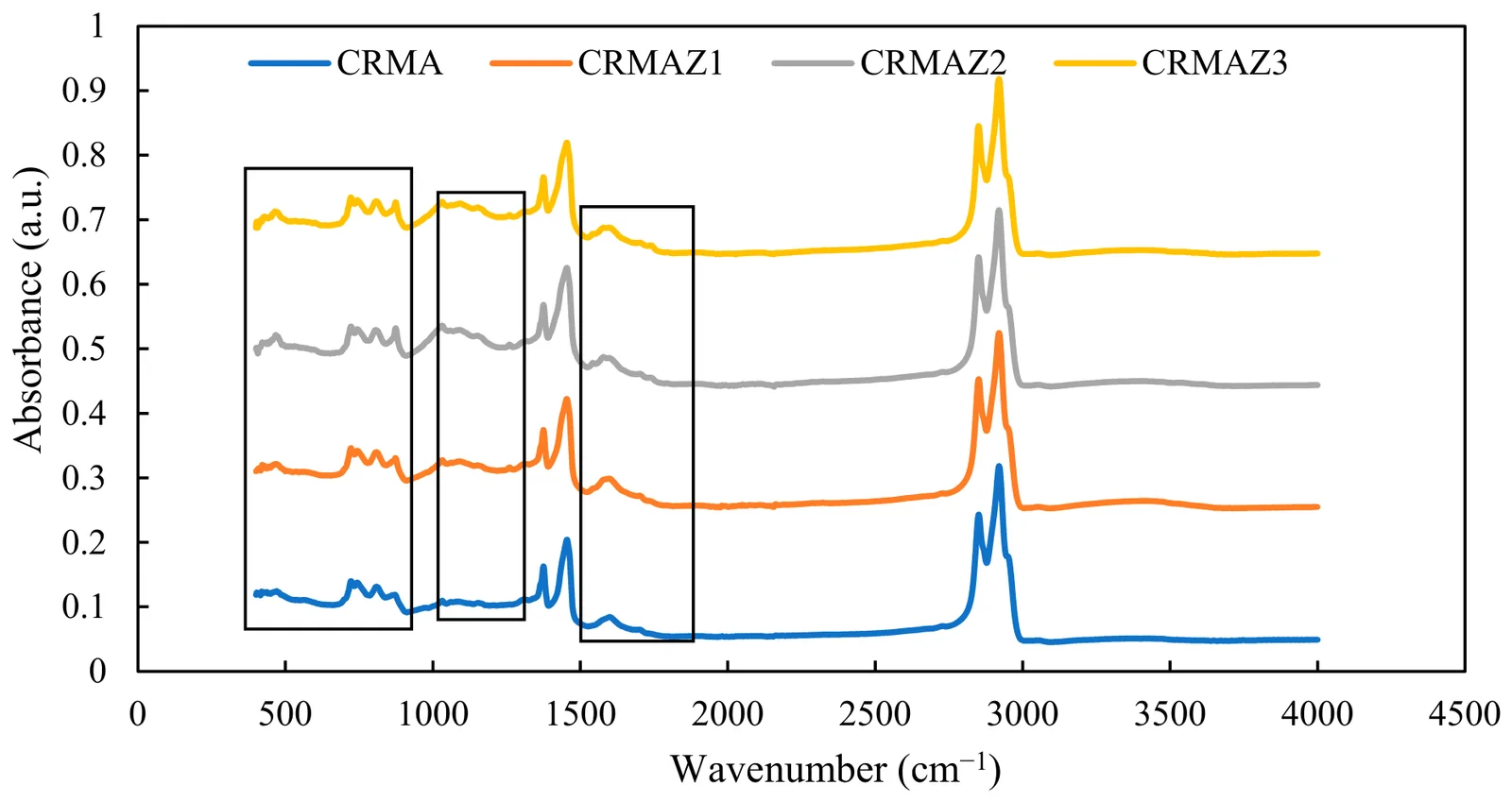
FTIR spectra of CRMAZ-series samples.

FTIR was employed as the first-stage physicochemical characterization to identify changes associated with crumb rubber and the warm-mix additives. To facilitate visual comparison, the spectra in Figure 4, Figure 5 and Figure 6 were vertically offset. Consequently, the absolute ordinate positions and unnormalized peak intensities were not used for quantitative comparison, and no quantitative band indices were calculated. All binders exhibited the principal hydrocarbon-related bands at approximately 2920 and 2850 cm^−1^, assigned to the asymmetric and symmetric stretching vibrations of aliphatic –CH_2_ groups, respectively. The bands at approximately 1454 and 1374 cm^−1^ were associated with –CH_2_ and –CH_3_ bending vibrations, while the band near 722 cm^−1^ was assigned to the rocking vibration of long-chain methylene groups. CRMA showed reproducible qualitative differences from QP in the fingerprint region, particularly around 722–807 cm^−1^, while no distinct new absorption band was resolved. The retained principal bands and absence of prominent new bands suggest that rubber modification was dominated by changes in component distribution and physical interaction rather than the formation of abundant new functional groups. The fingerprint-region differences provide preliminary evidence of changes in the local physicochemical environment associated with asphalt–rubber interaction; this interpretation is further supported by the subsequent rheological results.

The CRMAL-series spectra retained the same principal absorption bands as CRMA, and no distinct additive-induced band was resolved. This pattern supports the interpretation that the wax-based additive mainly altered the physical organization of the binder rather than generating abundant new chemical functionalities. When considered together with the known crystallization behavior of wax-based additives and the subsequent stiffness-oriented rheological response, the FTIR observations are consistent with the formation of a wax-associated physical structure. The CRMAZ-series spectra showed discernible qualitative differences in the fingerprint region, including the region near 1030 cm^−1^, where overlapping S=O and *C*–O contributions may occur, suggesting a change in the oxygen-containing chemical environment. Together with the subsequent increases in *γ*_c_ and tan*δ* and the changes in LAS and BBR indicators, these differences support a possible regulation of the continuous-phase composition and asphalt–rubber interface by the bio-oil-based additive. Accordingly, the FTIR spectra were used for qualitative band identification and interpreted in conjunction with the rheological evidence; quantitative ranking was not based on peak intensity.

### 3.2. Phase Behavior and Relaxation Characteristics

#### 3.2.1. Strain Dependence and Network-Structure Stability

**Figure 7:**
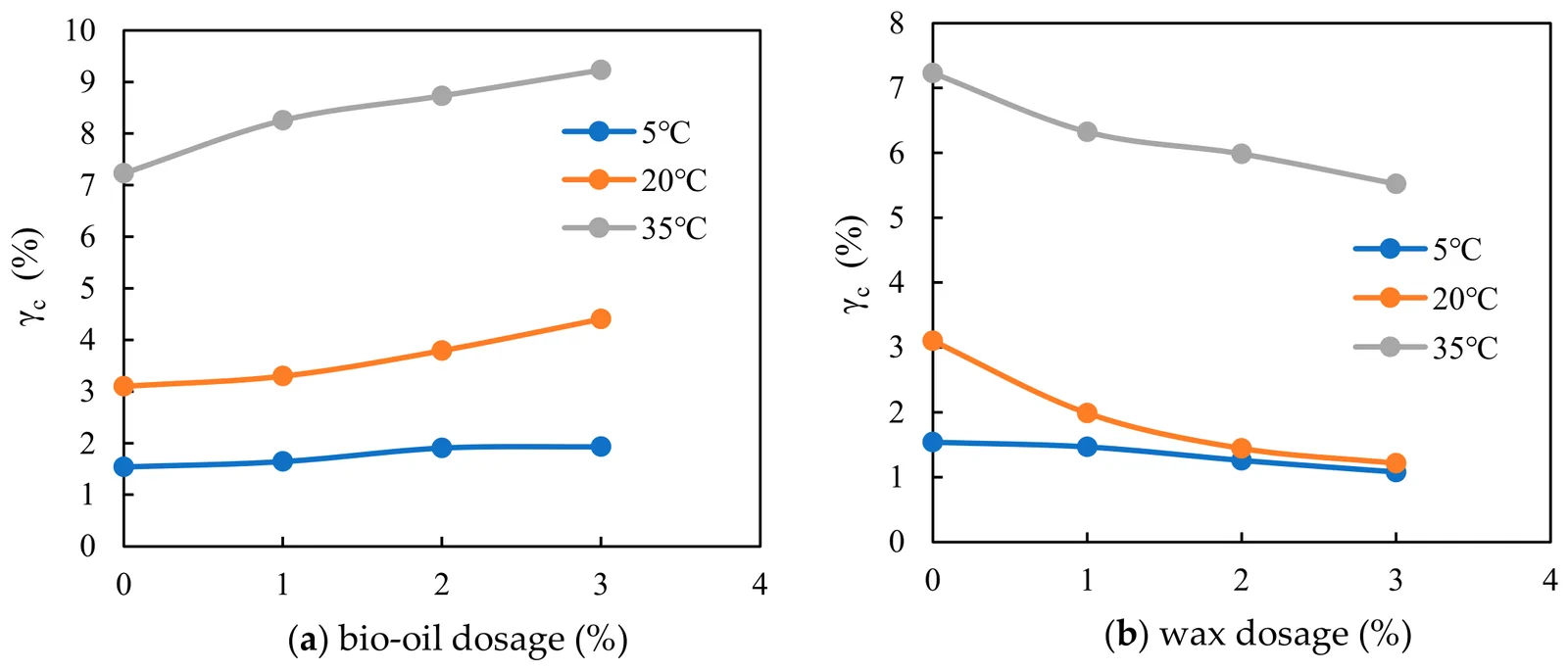
Variation in *γ*_c_ of different samples with temperature, warm-mix additive type, and dosage.

**Figure 8:**
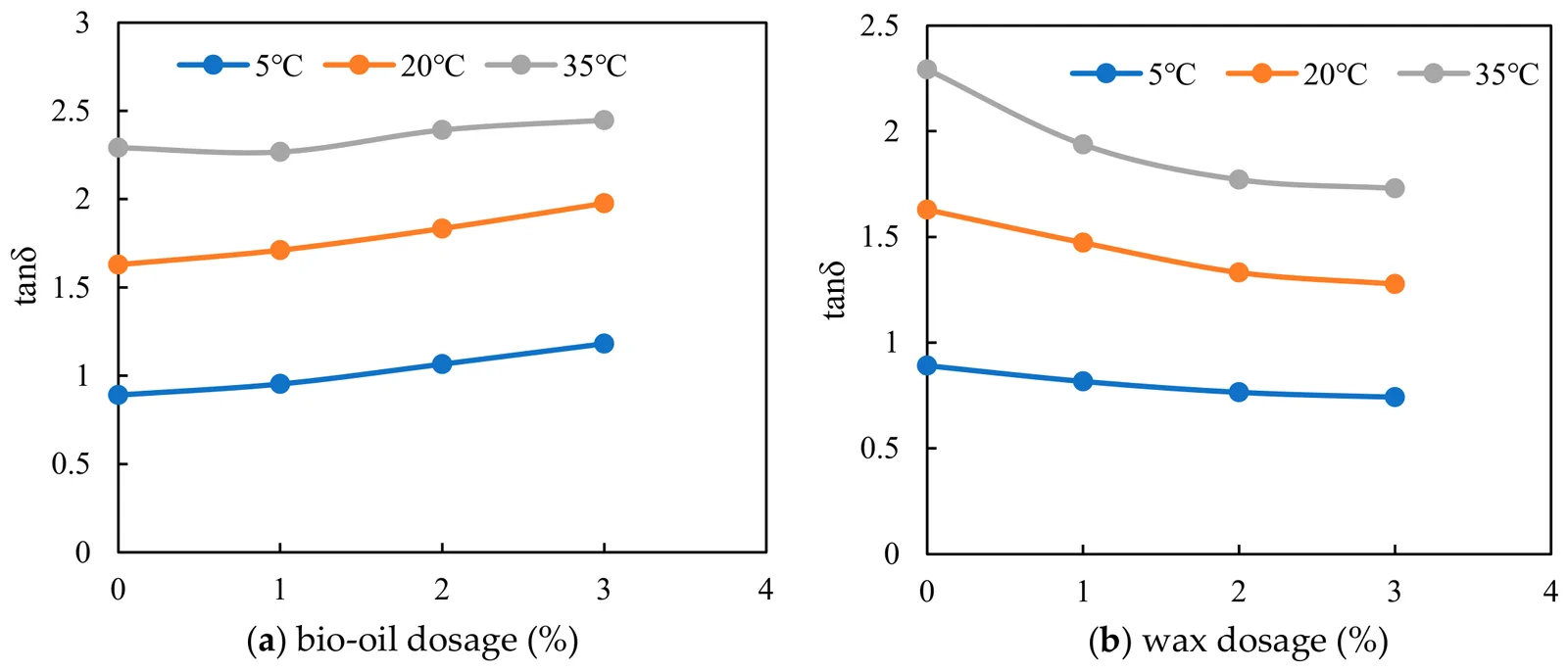
Variation in tan*δ* of different samples with temperature, warm-mix additive type, and dosage.

**Figure 9:**
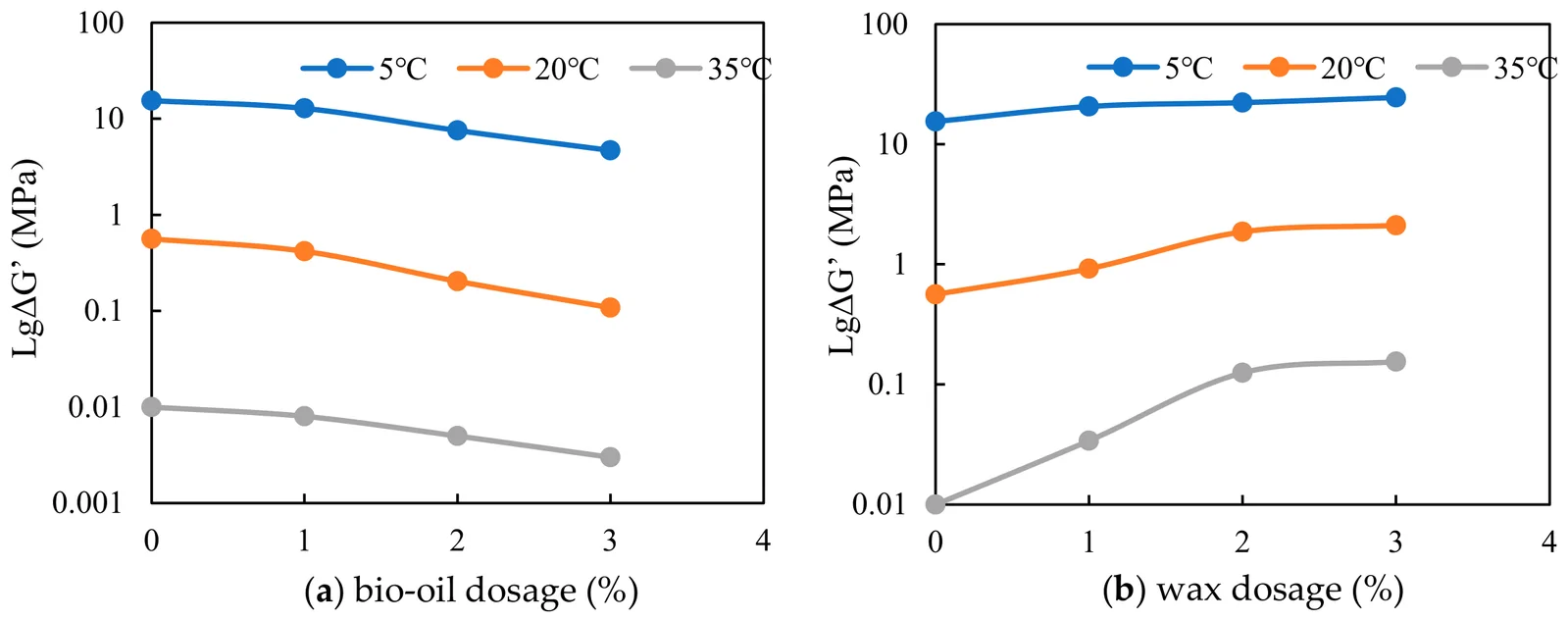
Variation in Δ*G*′ of different samples with temperature, warm-mix additive type, and dosage.

The strain sweep results in Figure 7, Figure 8 and Figure 9 can be used to evaluate the structural stability of binders within the LVE region and the network attenuation characteristics under large strain. *γ*_c_ represents the critical strain at which storage modulus *G*′ begins to deviate clearly from the plateau region; a larger value indicates a stronger ability to maintain structural stability from small to moderate deformation. tan*δ* reflects the relative relationship between viscous and elastic responses; a higher tan*δ* indicates stronger viscous dissipation and stress relaxation capacity. The Δ*G*′ value represents the absolute reduction in storage modulus after entering the nonlinear region and was used to characterize the extent of structural rearrangement or disruption of the rubber–asphalt network under large deformation. Because Δ*G*′ is an absolute quantity, it was interpreted together with the initial plateau stiffness and the accompanying *γ*_c_ and tan*δ* responses, rather than used as the sole ranking metric among binders with substantially different initial stiffnesses. For typical multiphase binders such as rubber-modified asphalt, synchronous changes in *γ*_c_, tan*δ*, and Δ*G*′ can reflect the combined influence of warm-mix additives on structural stability, relaxation capacity, and large-deformation network response.

As shown in Figure 7, Figure 8 and Figure 9, the two warm-mix additives produced different strain-dependent binder responses. For the CRMAZ series, *γ*_c_ and tan*δ* increased with the dosage, whereas Δ*G*′ decreased, indicating a wider LVE response range, greater viscous dissipation, and a more relaxation-oriented large-strain response. A plausible explanation is that the light and oxygen-containing polar components of the bio-oil-based additive enter the maltene phase and asphalt–rubber interfacial region, supplementing light components consumed during rubber swelling and reducing aggregation among asphaltene-rich structural units [35]. The resulting reduction in continuous-phase constraint may promote a more uniform strain redistribution and provides a coherent explanation for the combined *γ*_c_, tan*δ*, and Δ*G*′ trends.

The CRMAL series showed the opposite pattern: *γ*_c_ and tan*δ* decreased, while Δ*G*′ increased with dosage. These changes indicate earlier departure from the LVE region, a greater elastic or rigid contribution, and more pronounced modulus attenuation under large strain. This interpretation agrees with the reported crystallization behavior of wax-based additives [36,37]. Upon cooling, wax molecules may form microcrystalline domains that act as physical crosslinks and increase the small-strain elastic response. Once the imposed strain exceeds the capacity of this physical structure, disruption and rearrangement can produce a larger modulus reduction. Thus, wax-associated physical structuring provides a plausible explanation for the combined *γ*_c_, tan*δ*, and Δ*G*′ trends.

Taken together, the strain-sweep indicators distinguish a relaxation-oriented response for the CRMAZ series from a stiffness-oriented response for the CRMAL series. These contrasting viscoelastic states are relevant to the binder contribution to stress relaxation, deformation recovery, and cracking resistance in asphalt mixtures.

#### 3.2.2. Master Curves and Low-Frequency Quasi-Terminal Scaling

Master curves can shift frequency sweep results at different temperatures to a common reference temperature and are used to characterize the binder viscoelastic response over a wide frequency range. The high-frequency region corresponds to a short loading time or low-temperature conditions, whereas the low-frequency region corresponds to a long loading time or high-temperature conditions; therefore, the low-frequency response better reflects flow and relaxation characteristics under long-term loading. For an ideal homogeneous system, *G*′ and *G*″ in the low-frequency region usually approach scaling relationships of 2 and 1, respectively [26]. If the slopes deviate markedly from theoretical values, the system contains a more complex relaxation process or multiphase structural response. Master curves were constructed using 30 °C as the reference temperature (log(a_T_) = 0), and the shift factors at 5 °C and 55 °C were obtained by horizontal shifting. The quality of horizontal shifting was evaluated based on the smooth continuity and overlap of the shifted curves across different temperatures. Because only three testing temperatures (5 °C, 30 °C, and 55 °C) were employed in this study, WLF coefficients were not further fitted to avoid overinterpretation from limited temperature points. The obtained shift factors were directly used for constructing the master curves, as listed in Table 4.

**Figure 10:**
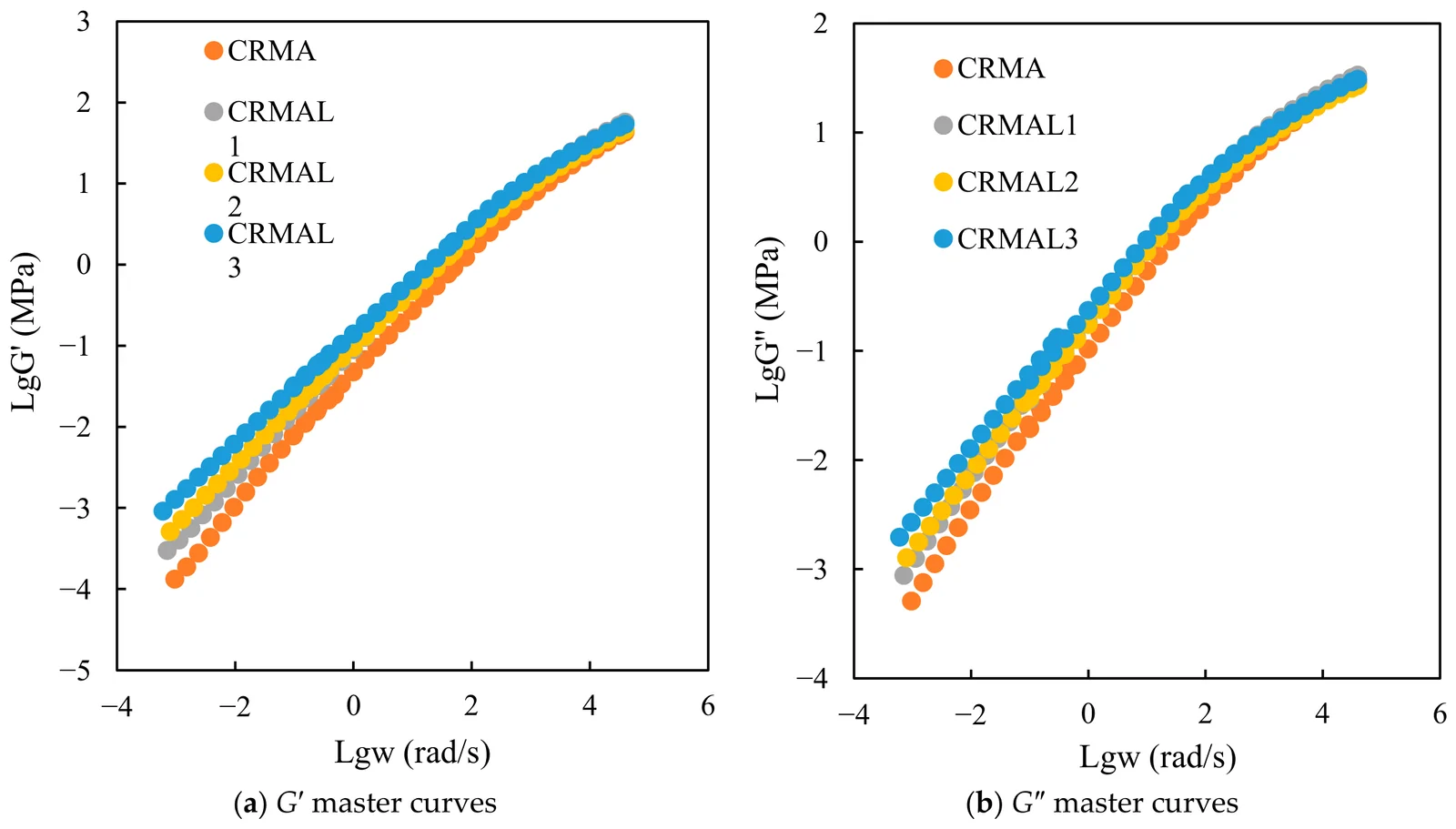
*G*′ and G″ master curves of wax-based warm-mix rubber-modified asphalt.

**Figure 11:**
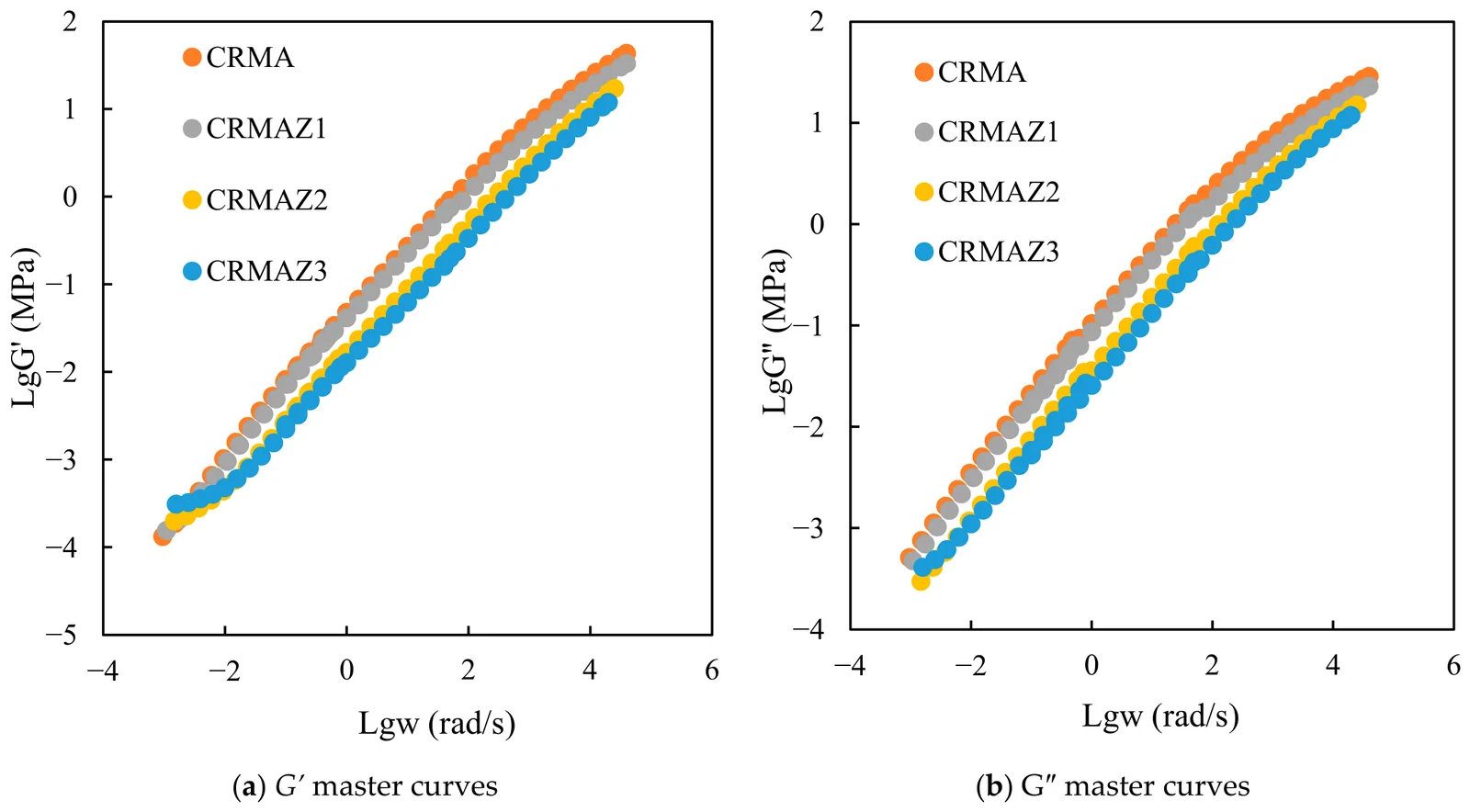
*G*′ and G″ master curves of bio-oil-based, warm-mix rubber-modified asphalt.

Figure 10 and Figure 11 show that both warm-mix systems formed relatively continuous *G*′ and *G*″ master curves, indicating a certain degree of time–temperature equivalence within the test range. However, differences among samples were more pronounced in the low-frequency region, suggesting that warm-mix additives mainly influenced the relaxation behavior of the rubber–asphalt system over long time scales. Although branching and dispersion were observed in the Black diagrams, indicating deviations from ideal thermorheological simplicity, horizontal shifting was still applied as an engineering approximation to compare the broadband viscoelastic response of different binders within the investigated temperature range. Therefore, the obtained master curves should be interpreted as comparative representations rather than exact descriptions of perfectly thermorheologically simple systems.

The low-frequency slopes were determined by linear regression within the low-frequency region of the master curves using seven consecutive points from the 55 °C dataset. The corresponding reduced-frequency intervals, expressed as lg (reduced angular frequency/[rad/s]), were −2.7139 to −1.5139 for QP, −3.0190 to −1.8190 for CRMA, −3.1490 to −1.9490 for CRMAL1, −3.0990 to −1.8990 for CRMAL2, −3.2200 to −2.0200 for CRMAL3, −2.9590 to −1.7590 for CRMAZ1, −2.8290 to −1.6290 for CRMAZ2, and −2.7990 to −1.5990 for CRMAZ3. Due to the multiphase nature of rubber-modified asphalt and the broad relaxation spectrum introduced by rubber particles, the observed low-frequency response should be regarded as a quasi-terminal region rather than a fully developed terminal region.

**Figure 12:**
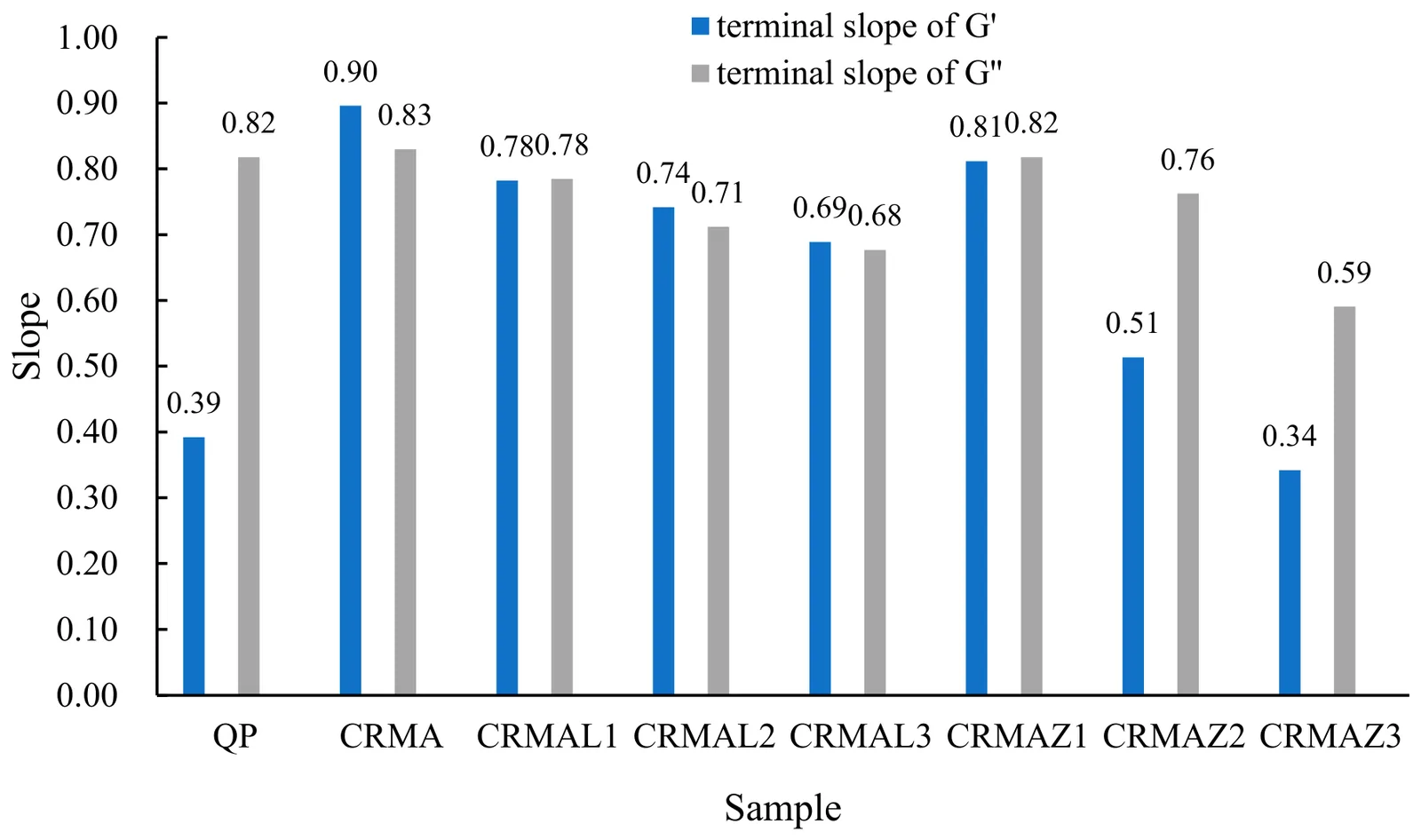
Low-frequency quasi-terminal slopes of *G*′ and G″ master curves for each asphalt binder.

Figure 12 presents the low-frequency quasi-terminal slopes of the *G*′ and G″ master curves. The *G*′ slopes of all samples were lower than the theoretical value of 2. The G″ slopes of QP and CRMA were approximately 0.82 and 0.83, respectively; the CRMAL-series values decreased from 0.78 to 0.68 with the dosage, while the CRMAZ-series values decreased from 0.82 to 0.59. Because the measured response represents a quasi-terminal region and horizontal shifting is an engineering approximation for these multiphase binders, the slopes provide a comparative measure of relaxation-spectrum dispersion and long-time rheological response.

#### 3.2.3. Analysis of Black and Cole–Cole Diagrams

**Figure 13:**
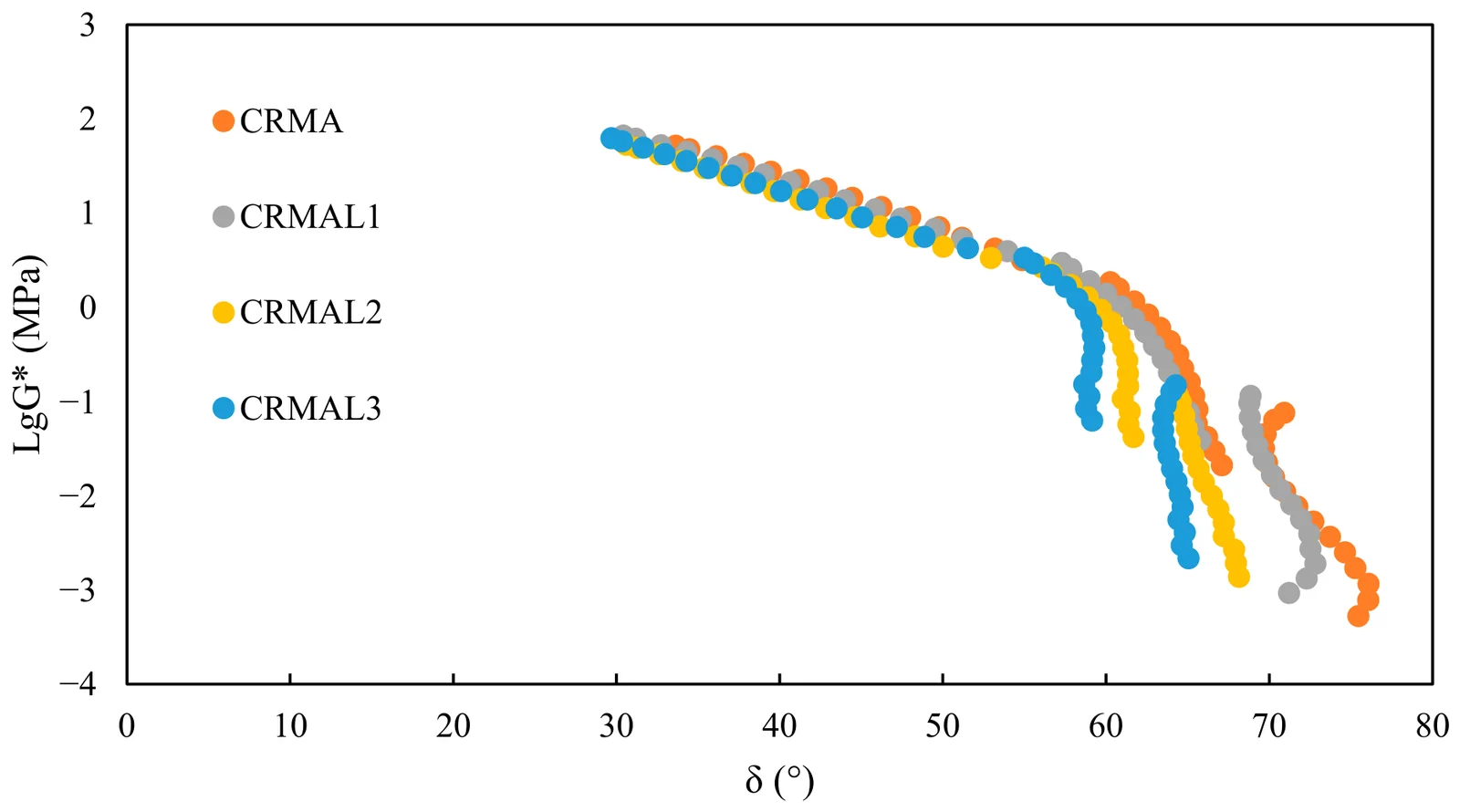
Black diagrams of wax-based, warm-mix rubber-modified asphalt samples.

**Figure 14:**
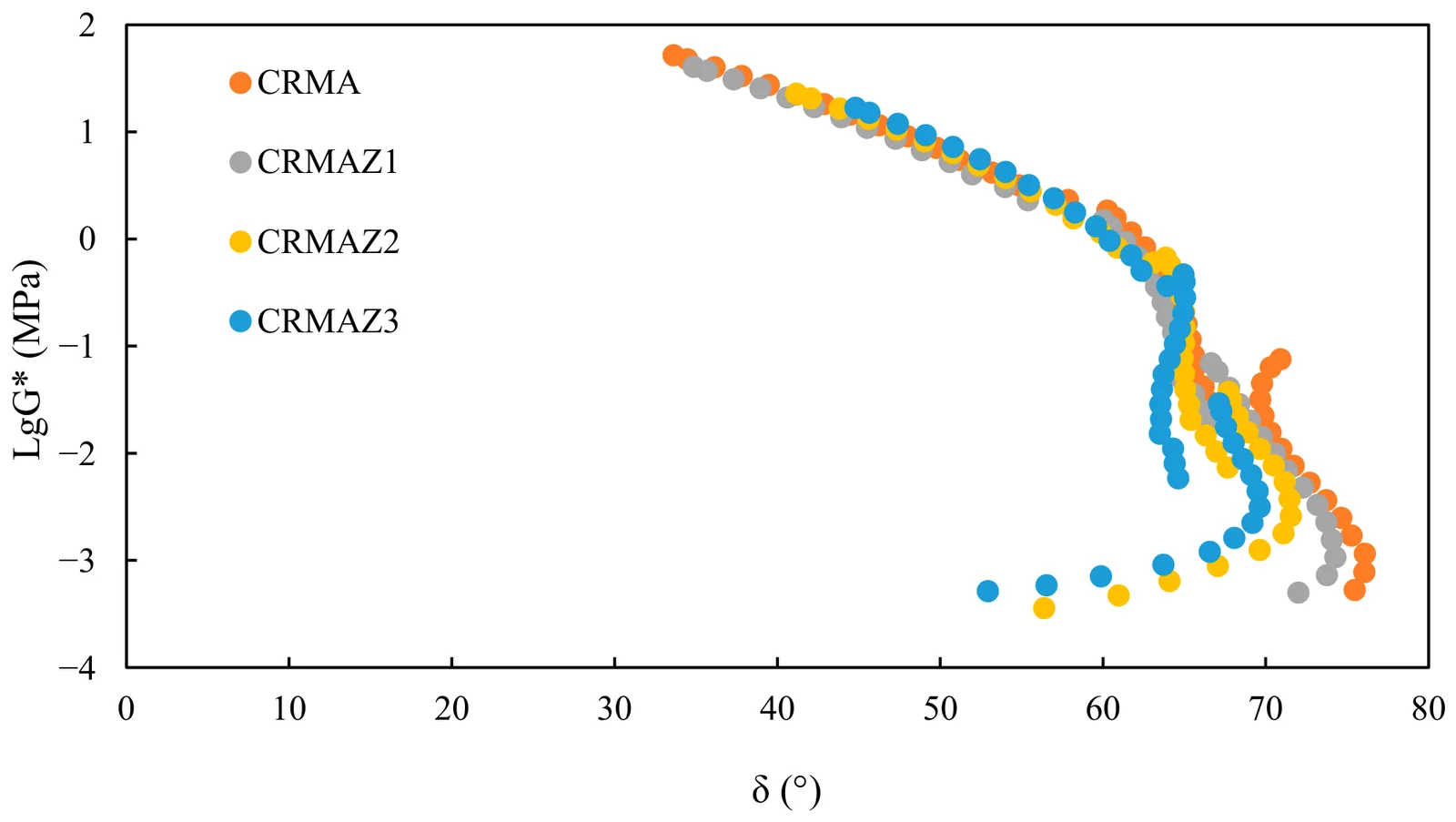
Black diagrams of bio-oil-based, warm-mix rubber-modified asphalt samples.

Black diagrams were employed to determine whether viscoelastic responses under different temperatures and frequencies could be merged into a single curve. Figure 13 and Figure 14 show that the data points did not completely form a single smooth curve, but exhibited different degrees of dispersion and branching in the high-phase-angle region, indicating multiple relaxation characteristics. With an increasing wax-based additive dosage, the CRMAL series showed greater dispersion, particularly in the medium-to-high phase-angle region, consistent with increased response heterogeneity among structural units. The CRMAZ series remained close to CRMA at a low dosage, whereas more pronounced branching at a higher dosage suggested a broader or more complex relaxation response.

**Figure 15:**
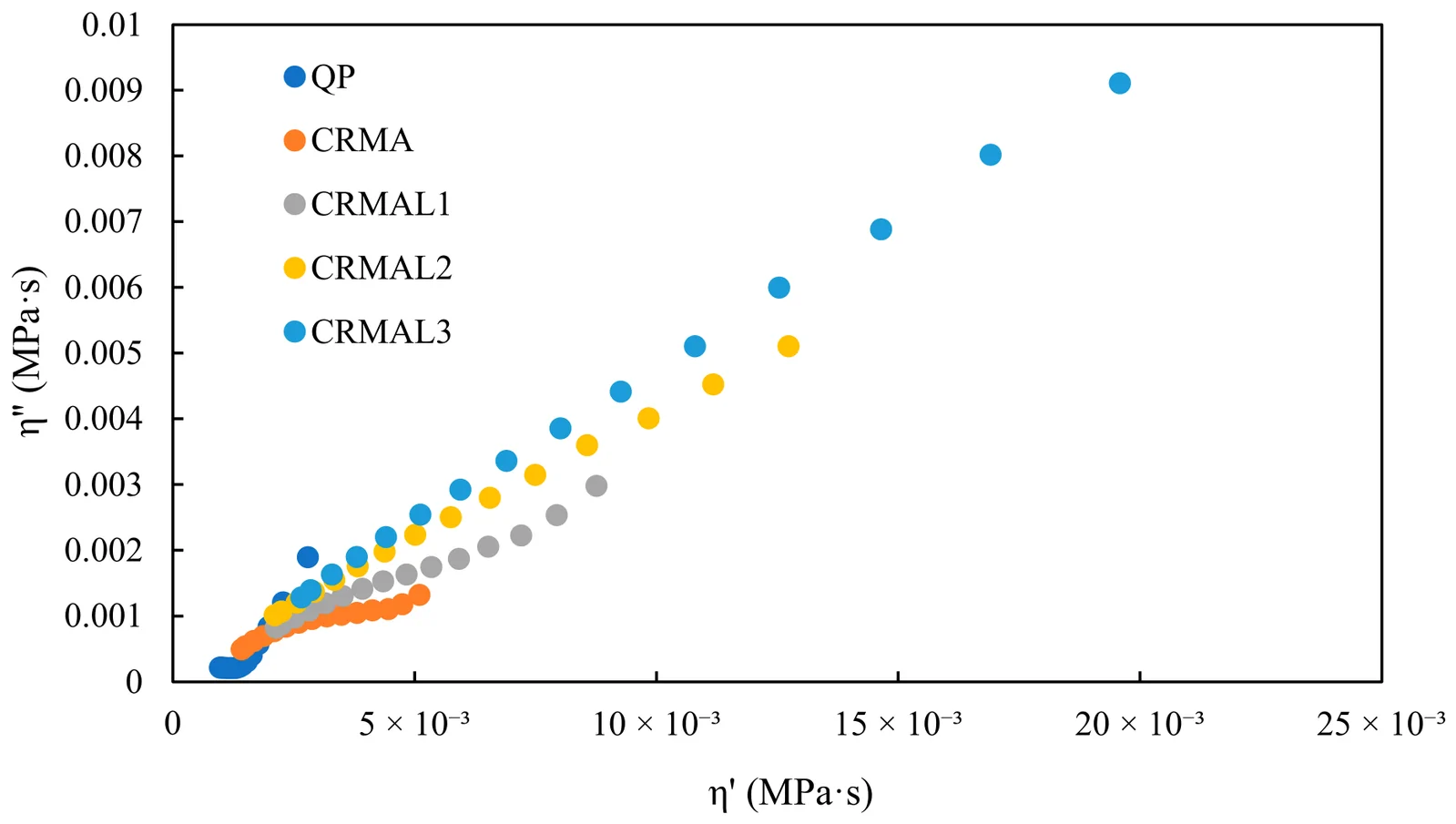
Cole–Cole diagrams of wax-based, warm-mix rubber-modified asphalt samples.

**Figure 16:**
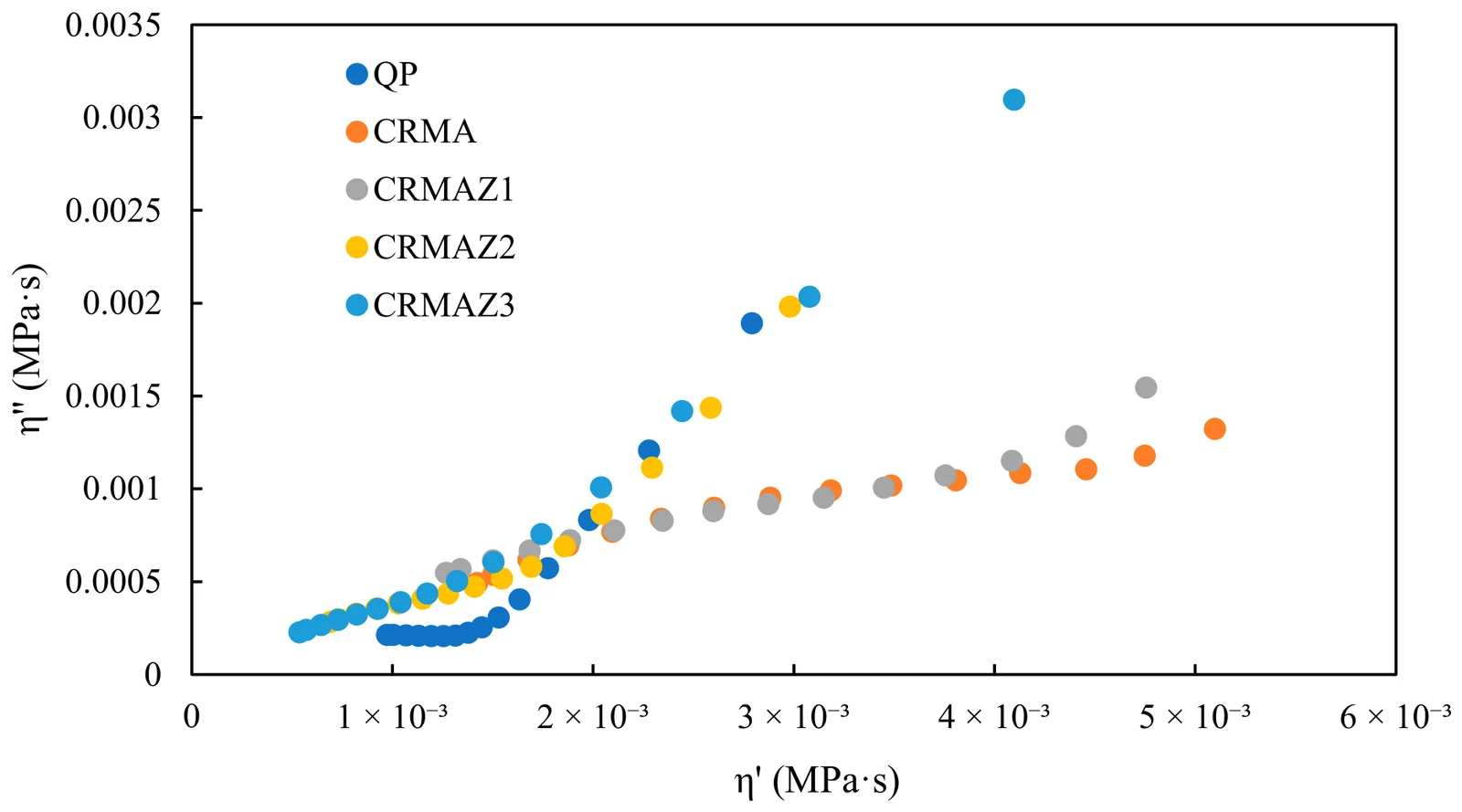
Cole–Cole diagrams of bio-oil-based, warm-mix rubber-modified asphalt samples.

Cole–Cole diagrams further reflect the relaxation-time distribution of binders. In general, a more concentrated trajectory indicates a narrower relaxation distribution, whereas outward movement or greater spreading indicates a broader distribution. As shown in Figure 15 and Figure 16, the CRMA trajectory shifted outward relative to QP. The CRMAL series showed progressively greater outward movement and expansion with the dosage, while the CRMAZ trajectories were comparatively less expanded; CRMAZ1 remained close to CRMA, and CRMAZ2 and CRMAZ3 shifted outward to a smaller extent than the wax-based series. Within the present descriptive comparison, these curve patterns are consistent with a broader relaxation dispersion for CRMAL and a comparatively more relaxation-oriented, less rigidly constrained response for CRMAZ.

Collectively, the strain-sweep, master-curve, Black-diagram, and Cole–Cole results show that the two additives produced different stiffness and relaxation responses. The CRMAL series showed greater rigidity and broader relaxation dispersion with the dosage, whereas the CRMAZ series showed greater viscous relaxation but also increased low-frequency dispersion at a high dosage. Together, these analyses establish a consistent rheological contrast between the two additive systems; the underlying mechanisms are integrated in Section 4.

### 3.3. High-Temperature Deformation and Recovery Response (MSCR)

**Figure 17:**
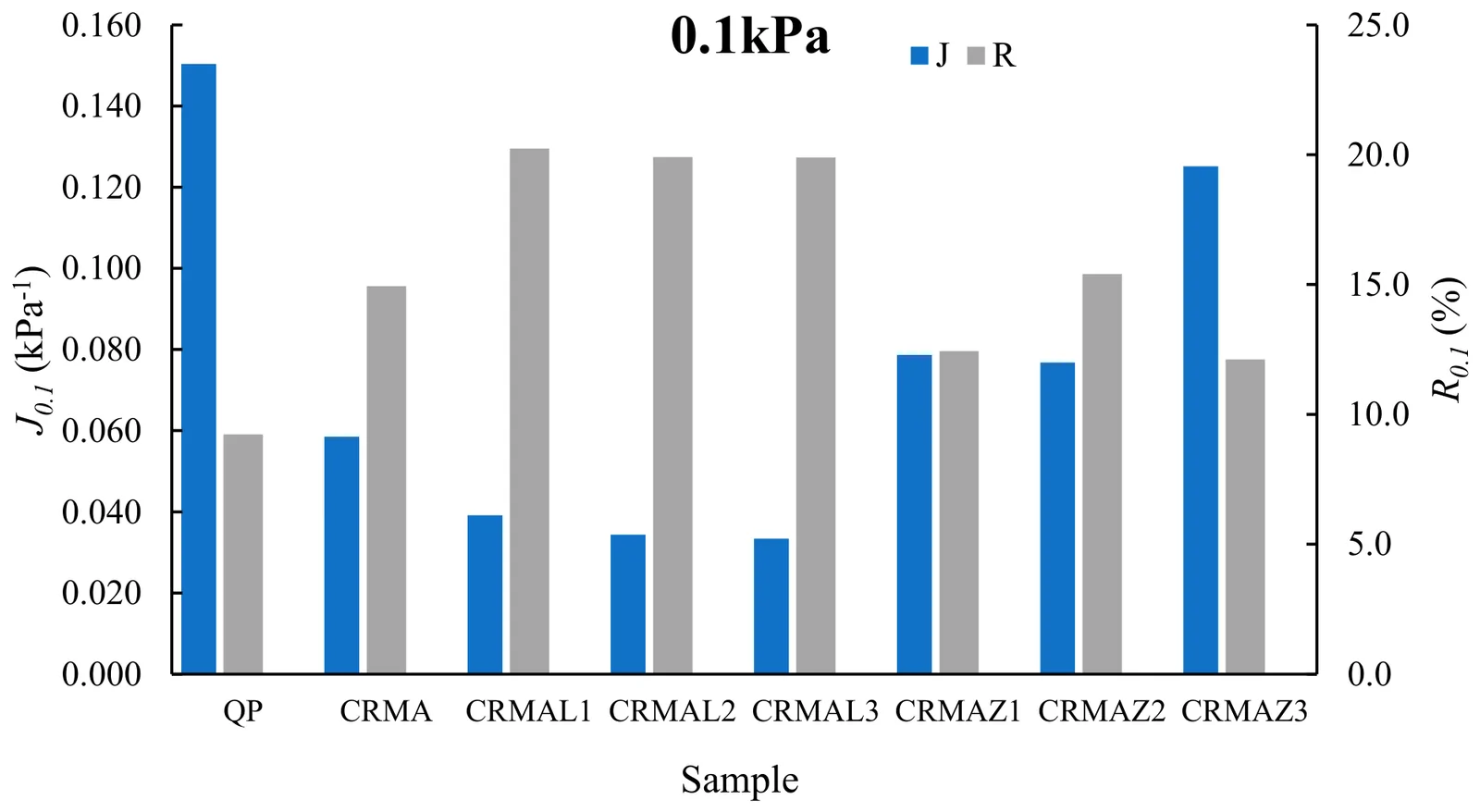
*J*_nr_ and *R* values of asphalt samples under 0.1 kPa stress.

**Figure 18:**
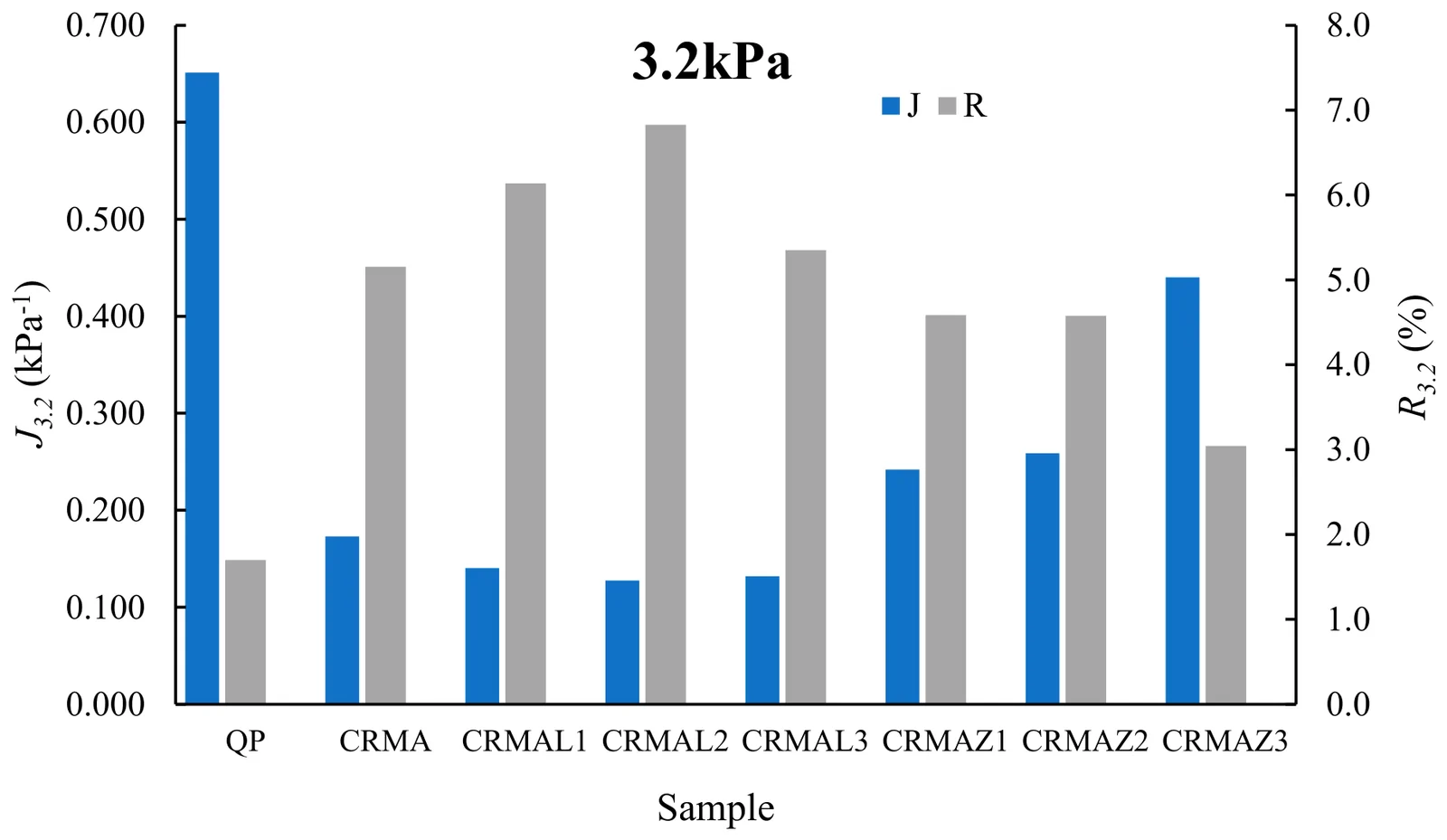
*J*_nr_ and *R* values of asphalt samples under 3.2 kPa stress.

The MSCR test was employed to compare high-temperature deformation and recovery at 58 °C. Figure 17 and Figure 18 show *J*_nr_ and *R* at 0.1 and 3.2 kPa. Lower *J*_nr_ and higher *R* indicate greater resistance to nonrecoverable deformation and stronger elastic recovery, both of which are important binder contributions to the rutting resistance of asphalt mixtures. *J*_nr_ is reported in kPa^−1^ and *R* in percent.

At 0.1 kPa, QP exhibited the highest *J*_nr_ (approximately 0.150 kPa^−1^) and the lowest *R* (9.23%). CRMA showed a lower *J*_nr_ of approximately 0.058 kPa^−1^ and a higher *R* of 14.94%. The CRMAL series further reduced *J*_nr_ to approximately 0.033–0.039 kPa^−1^ and showed *R* values of 19.89–20.24%. In contrast, the CRMAZ series generally exhibited higher *J*_nr_ than CRMA, and CRMAZ3 reached approximately 0.125 kPa^−1^. The CRMAL response therefore indicates a stronger contribution to high-temperature deformation resistance and recovery at low stress.

Under 3.2 kPa, *J*_nr_ increased and *R* decreased for all binders relative to 0.1 kPa. QP had the highest *J*_nr_ (approximately 0.651 kPa^−1^) and lowest *R* (1.70%), whereas CRMA had *J*_nr_ of approximately 0.173 kPa^−1^ and *R* of 5.16%. The CRMAL series showed *J*_nr_ values of 0.128–0.140 kPa^−1^ and *R* values of 5.35–6.83%; CRMAL2 combined the lowest *J*_nr_ (approximately 0.128 kPa^−1^) with the highest *R* (6.83%). In the CRMAZ series, *J*_nr_ increased from approximately 0.242 to 0.440 kPa^−1^, and *R* decreased from approximately 4.59% to 3.04% with the dosage, indicating a progressively less favorable high-stress binder deformation-and-recovery balance at a higher bio-oil dosage.

Table 5 further shows that the *J*_nr,diff_ and *R*_diff_ values of QP were 333.0% and 81.6%, respectively. For CRMA, the corresponding values were 196.0% and 65.5%, which were lower than those of QP but still indicated pronounced stress dependence. All binders exhibited *J*_nr,diff_ values above 75%, indicating that the absolute stress sensitivity remained high and that the indices should be interpreted comparatively rather than as evidence of full stress stability. Within the CRMAL series, *J*_nr,diff_ increased from 258.7% to 295.2% with the dosage, while *R*_diff_ ranged from 65.7% to 73.1%; CRMAL2 retained the lowest *R*_diff_ within this series. For the CRMAZ series, *J*_nr,diff_ increased from 207.6% to 251.7%, and *R*_diff_ increased from 63.1% for CRMAZ1 to 74.9% for CRMAZ3, indicating that excessive bio-oil-based warm-mix additive increased high-stress sensitivity.

Considering *J*_nr_, *R*, and the stress-sensitivity indices together, CRMAL2 showed the most favorable combination of high-temperature deformation and recovery indicators among the tested modified binders. This balance suggests that the intermediate wax dosage provides the strongest binder contribution to rutting resistance, whereas an increasing bio-oil dosage progressively weakens high-stress recovery.

### 3.4. Intermediate-Temperature Fatigue-Related Response (LAS)

**Figure 19:**
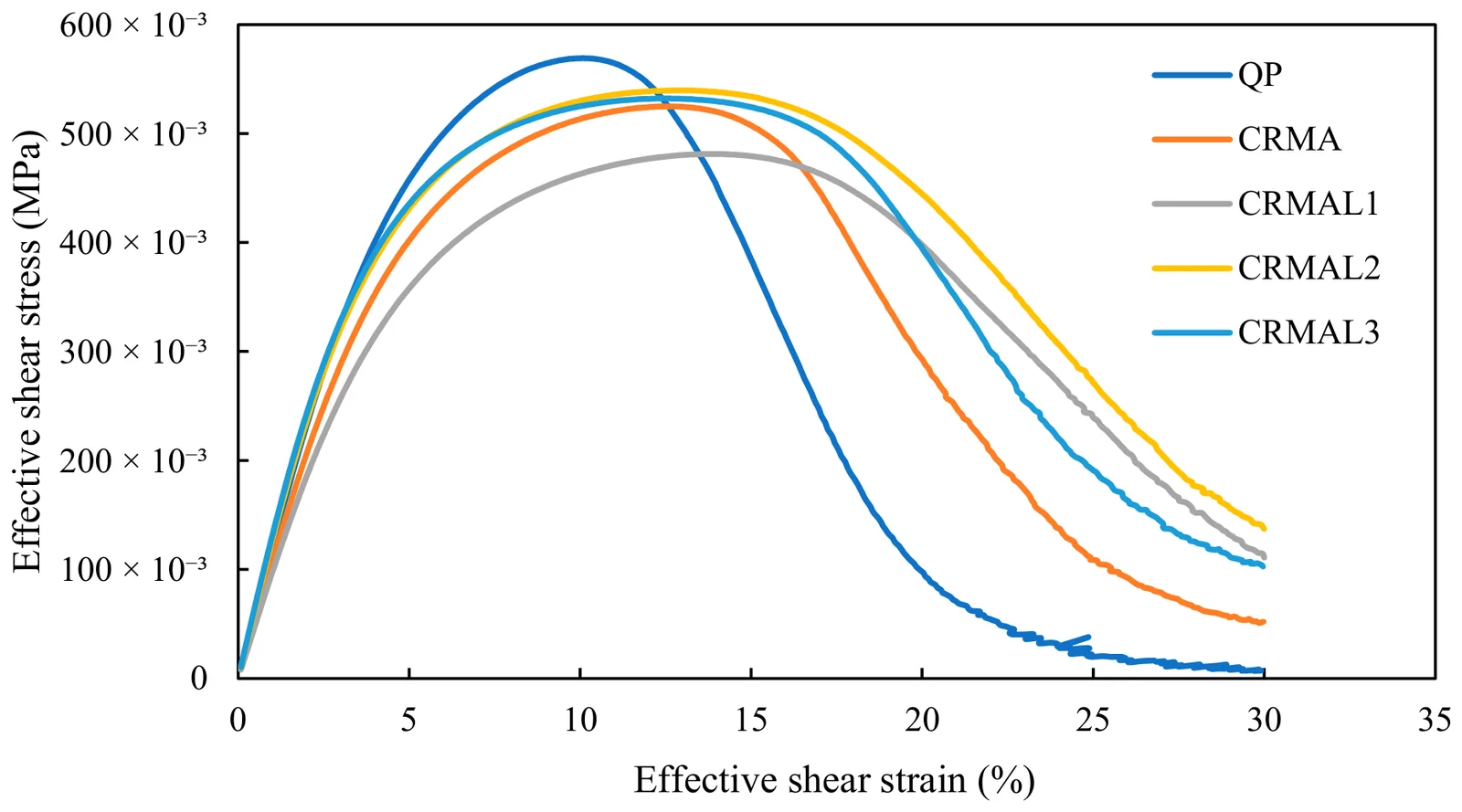
Stress–strain curves of wax-based, warm-mix rubber-modified asphalt.

**Figure 20:**
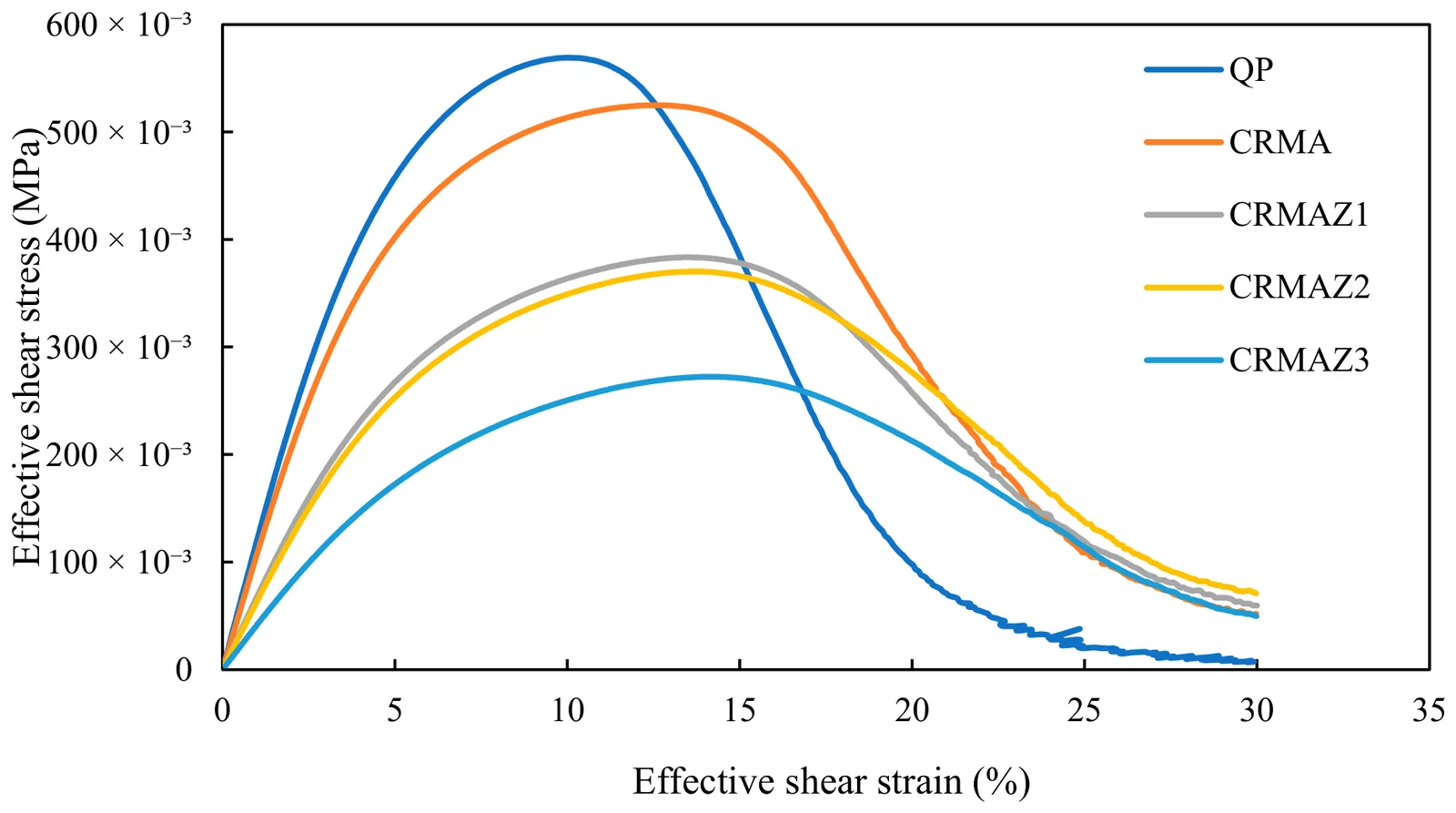
Stress-strain curves of bio-oil-based, warm-mix rubber-modified asphalt.

The LAS test was employed to compare fatigue-related damage evolution and predicted *N_f_* under intermediate-temperature conditions. Figure 19 and Figure 20 show the effective stress–strain curves of the wax-based and bio-oil-based systems. QP reached its peak at a lower strain and decayed more rapidly than CRMA, indicating faster damage development under the LAS loading protocol. The stress–strain response and VECD-based *N_f_* provide established rheological indicators of the binder contribution to fatigue-cracking resistance.

For the wax-based system, the CRMAL curves generally extended to higher strain than CRMA. CRMAL1 and CRMAL2 showed slower post-peak attenuation, whereas CRMAL3 decreased more rapidly after the peak, suggesting a dosage-related trade-off associated with increased rigidity. For the bio-oil-based system, lower peak stress and longer curve extension in the medium-to-high strain region were observed, especially for CRMAZ2 and CRMAZ3. These curve features are consistent with a reduced stress concentration and greater relaxation-oriented deformation capacity, thereby helping delay fatigue-damage accumulation.

**Figure 21:**
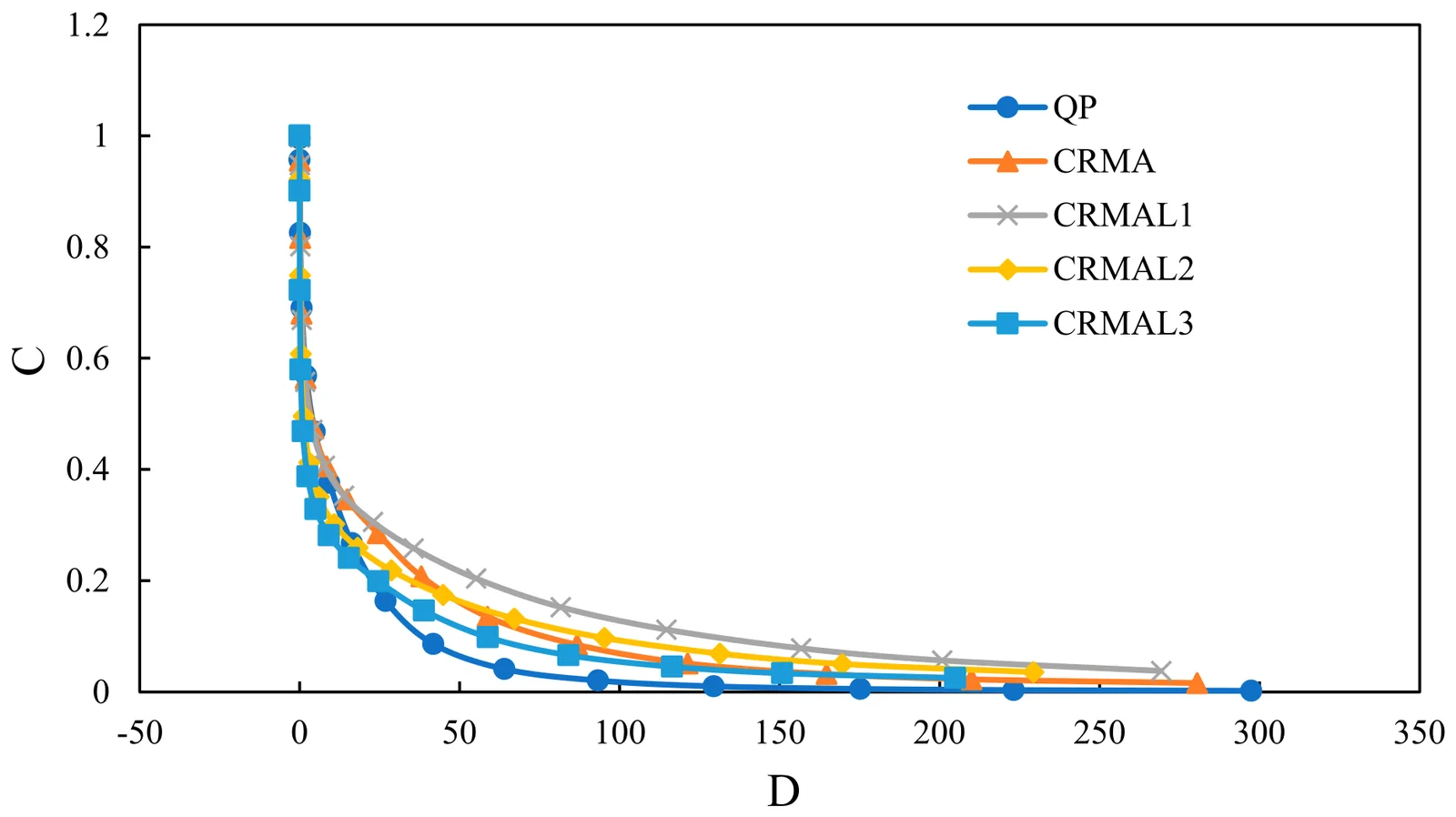
*C*-*D* curves of wax-based, warm-mix rubber-modified asphalt.

**Figure 22:**
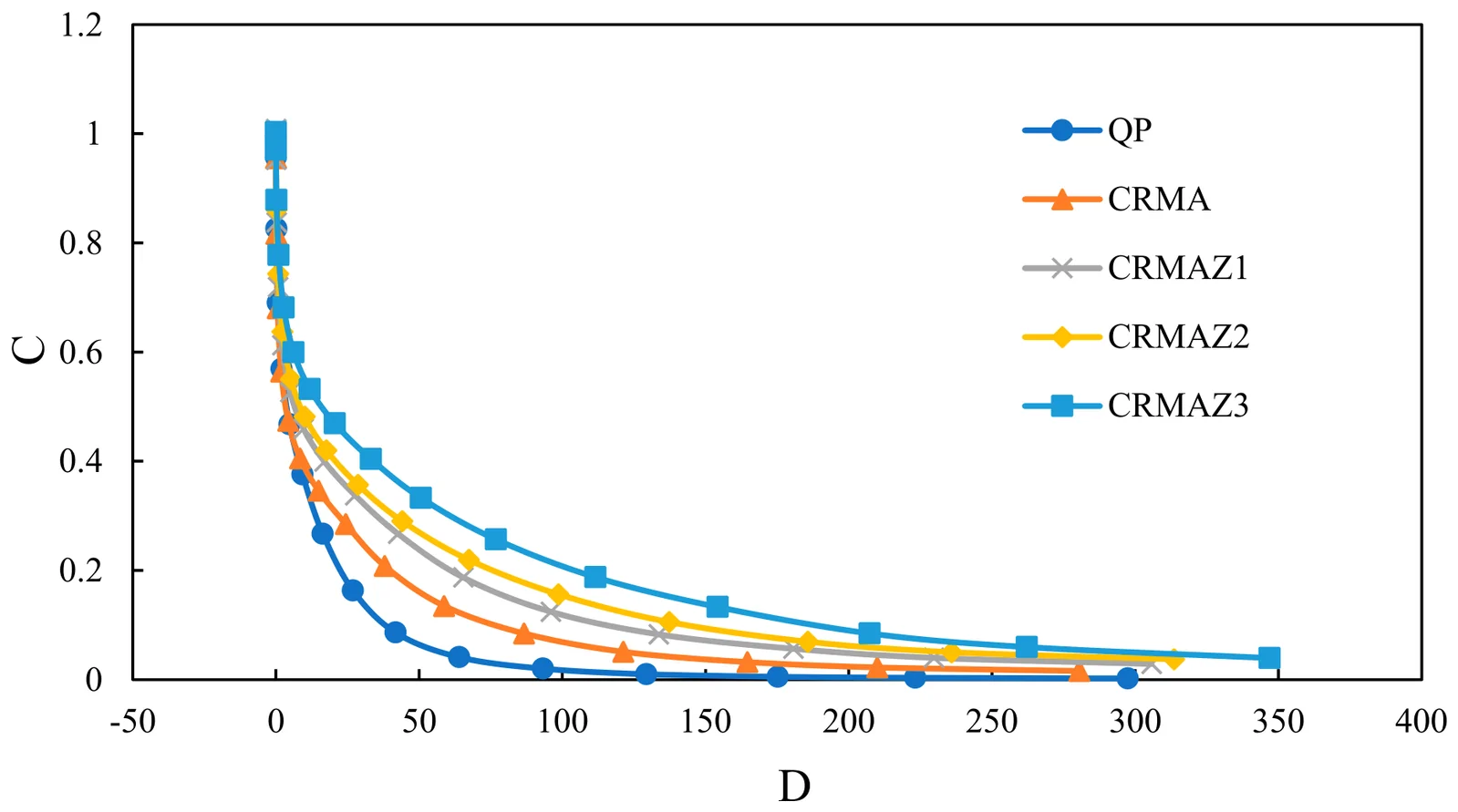
*C*-*D* curves of bio-oil-based, warm-mix rubber-modified asphalt.

Figure 21 and Figure 22 show the *C*-*D* damage characteristic curves obtained from the VECD model. QP showed the most rapid decrease in *C* with increasing *D*, while the CRMA curve shifted to the right. Within the CRMAL series, CRMAL1 and CRMAL2 were generally located to the right of CRMA, whereas the additional shift for CRMAL3 was limited. The CRMAZ curves showed progressively greater rightward displacement with the dosage, and CRMAZ3 maintained a higher *C* over a larger *D* range. Together with the corresponding *N_f_* values, these curve positions indicate a dosage-dependent fatigue-related response and a greater binder contribution to damage tolerance for the CRMAZ series.

**Figure 23:**
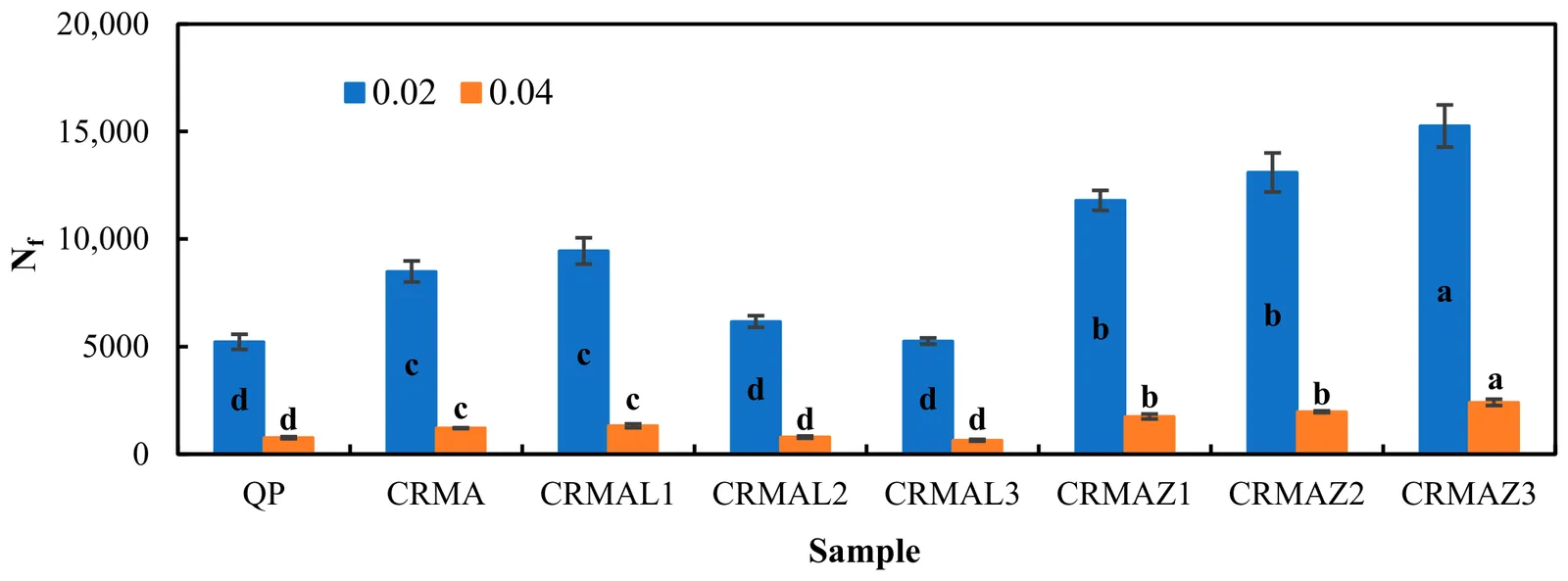
LAS-predicted binder *N_f_* values at 2% and 4% strains. Values are mean ± SD (*n* = 3); different lowercase letters indicate significant differences among binders at the same strain level (Tukey’s test, *p* < 0.05).

Figure 23 presents the LAS-predicted *N_f_* values at 2% and 4% strain as the mean ± SD (*n* = 3). One-way ANOVA identified a significant formulation effect at 2% strain (*F*(7,16) = 120.70, *p* < 0.001). Tukey’s test showed that all CRMAZ binders had higher *N_f_* than CRMA; CRMAZ3 was also higher than CRMAZ1 and CRMAZ2, whereas CRMAZ1 and CRMAZ2 did not differ significantly. CRMAL1 did not differ significantly from CRMA, while CRMAL2 and CRMAL3 were lower than CRMA (*p* < 0.05).

At 4% strain, the *N_f_* values decreased for all binders, and the formulation effect remained significant (*F*(7,16) = 191.10, *p* < 0.001). The same principal pairwise pattern was retained: all CRMAZ binders exceeded CRMA, CRMAZ3 exceeded CRMAZ1 and CRMAZ2, CRMAL1 did not differ significantly from CRMA, and CRMAL2 and CRMAL3 were lower than CRMA (Tukey’s test, *p* < 0.05).

Overall, the LAS results demonstrate a statistically supported, dosage-dependent response for the tested formulations. The CRMAZ series showed a significantly higher predicted *N_f_* than CRMA at both strain levels, with CRMAZ3 providing the highest value; the wax-based response was non-monotonic, and higher wax dosages reduced *N_f_*. These results support the greater binder contribution of the bio-oil-based additive to intermediate-temperature fatigue-damage tolerance within the LAS framework.

### 3.5. Low-Temperature Stiffness and Relaxation Response (BBR)

**Figure 24:**
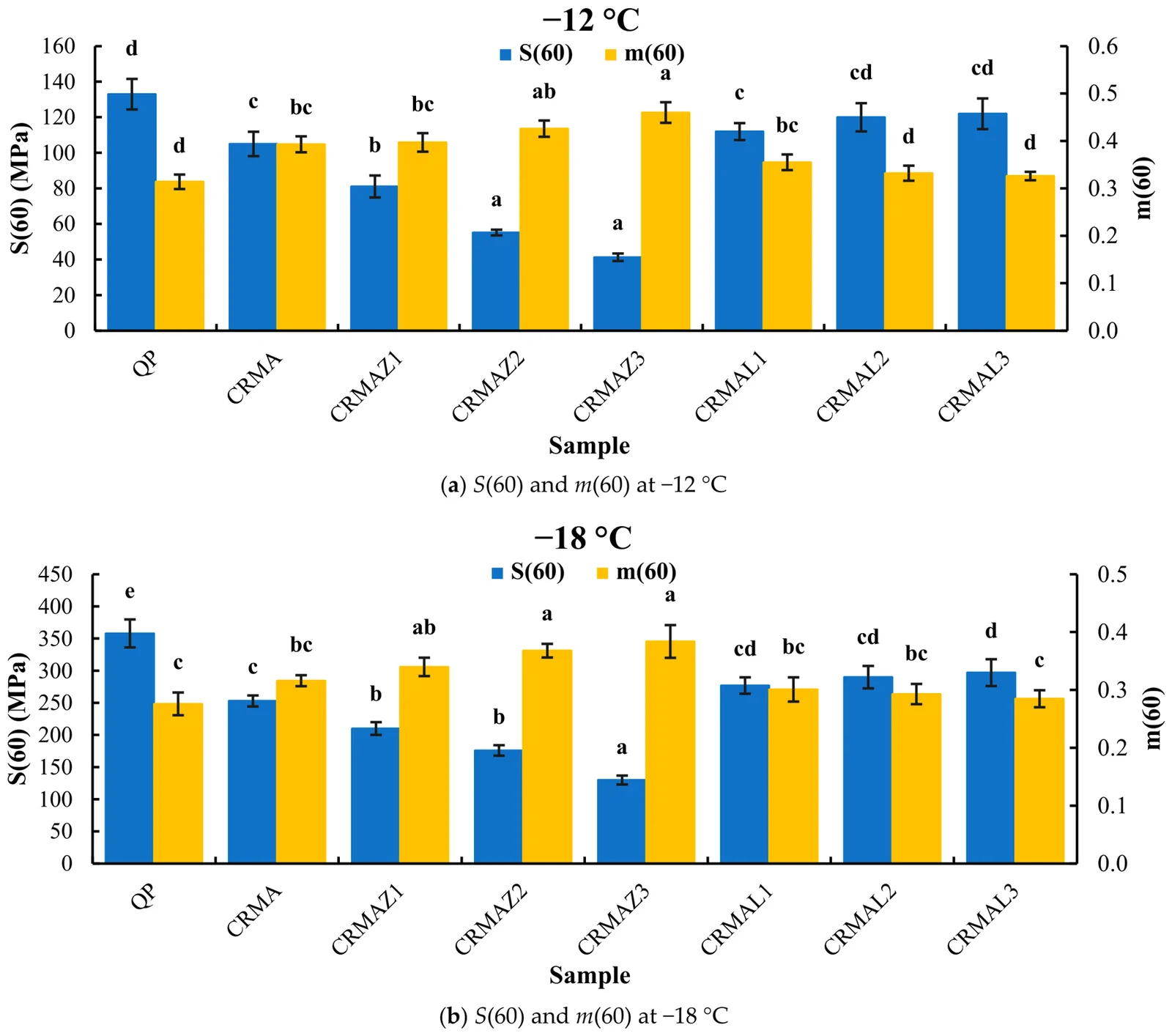
BBR test results: Values are mean ± SD (*n* = 3); different lowercase letters indicate significant differences among binders within the same temperature–indicator combination (Tukey’s test, *p* < 0.05). Groups sharing at least one letter are not significantly different.

The BBR results were used to compare low-temperature stiffness and stress relaxation, which govern the binder contribution to thermal-stress development and cracking resistance. Values are the mean ± SD from three replicate beam specimens, and the error bars in Figure 24 represent the SD calculated separately for each binder and test condition. One-way ANOVA showed significant formulation effects for *S*(60) and *m*(60) at both −12 °C (*F*(7,16) = 82.27 and 28.72, respectively; both *p* < 0.001) and −18 °C (*F*(7,16) = 77.53 and 14.41, respectively; both *p* < 0.001).

At −12 °C, QP had a higher *S*(60) and lower *m*(60), whereas CRMA showed a lower *S*(60) and higher *m*(60). Tukey’s test showed that all CRMAZ binders had a significantly lower *S*(60) than CRMA, whereas none of the CRMAL binders differed significantly from CRMA. For *m*(60), CRMAZ3 was significantly higher than CRMA, while CRMAZ1 and CRMAZ2 were not; CRMAL2 and CRMAL3 were significantly lower than CRMA (*p* < 0.05).

At −18 °C, *S*(60) increased and *m*(60) decreased for all binders. All CRMAZ binders had a significantly lower *S*(60) than CRMA; CRMAL1 and CRMAL2 did not differ significantly from CRMA, whereas CRMAL3 was significantly higher. For *m*(60), CRMAZ2 and CRMAZ3 were significantly higher than CRMA, while CRMAZ1 and all CRMAL binders did not differ significantly from CRMA (Tukey’s test, *p* < 0.05).

Taken together, the BBR results show that the relaxation-oriented CRMAZ response was supported most consistently by the stiffness comparisons at both temperatures, while the significant *m*(60) differences depended on dosage and temperature. CRMAZ3 combined the lowest *S*(60) with the highest *m*(60) at −18 °C. The wax-based series retained a more stiffness-oriented response, although several pairwise differences from CRMA were not statistically significant. The nonsignificant pairwise comparisons do not indicate an absence of an overall formulation effect: the omnibus ANOVA was significant, but the corresponding mean separations were small relative to the within-group SD, and *n* = 3 per condition limited the power to resolve modest pairwise effects. These comparisons were therefore interpreted as numerical trends rather than confirmed differences.

## 4. Discussion

Across the independent tests, the observed trends consistently distinguish a stiffness-oriented response for the wax-based additive from a relaxation-oriented response for the bio-oil-based additive. FTIR provides the initial physicochemical evidence, while the strain-sweep, master-curve, MSCR, LAS, and BBR indicators provide rheological corroboration. This cross-method agreement strengthens the internal consistency of the proposed mechanisms and is engineering-relevant because binder rheology strongly influences stress transfer, deformation recovery, and relaxation within asphalt mixtures. One-way ANOVA with Tukey’s multiple-comparison test confirmed formulation effects in the replicated LAS and BBR results. The DSR and MSCR outcomes provide complementary binder-level evidence of strain dependence, relaxation, deformation recovery, and stress sensitivity and are interpreted together with the statistically evaluated LAS and BBR indicators. Mixture behavior also depends on aggregate structure, volumetric composition, aging, construction, and environmental conditions.

For the wax-based system, the retained principal FTIR bands and absence of distinct new bands indicate a predominantly physical modification pathway. This observation, together with a reduced tan*δ*, increased Δ*G*′, broader relaxation dispersion, and comparatively lower *J*_nr_ and higher *R* at an intermediate dosage, is consistent with the wax-associated physical structuring and increased binder rigidity reported in previous studies [36,37]. The lower LAS-predicted *N_f_* and less favorable BBR stiffness–relaxation balance at a higher dosage reveal an engineering trade-off: a moderate wax content can strengthen high-temperature deformation recovery, whereas excessive structuring can reduce the relaxation capacity needed to resist fatigue and thermal cracking.

For the bio-oil-based system, the FTIR differences near 1030 cm^−1^ suggest a change in the oxygen-containing chemical environment. Considered together with an increased *γ*_c_ and tan*δ*, reduced Δ*G*′, higher LAS-predicted *N_f_*, and lower *S*(60) with higher *m*(60), these observations support regulation of the continuous-phase composition and asphalt–rubber interface as a plausible interpretation consistent with the previous literature [18,35]. This mechanism explains the improved relaxation, fatigue-related, and low-temperature responses, while the simultaneous increase in *J*_nr_ and decrease in *R* at a high dosage identify a trade-off in high-temperature deformation recovery. Microstructural and mixture testing can further quantify how this balance develops in asphalt mixtures.

**Figure 25:**
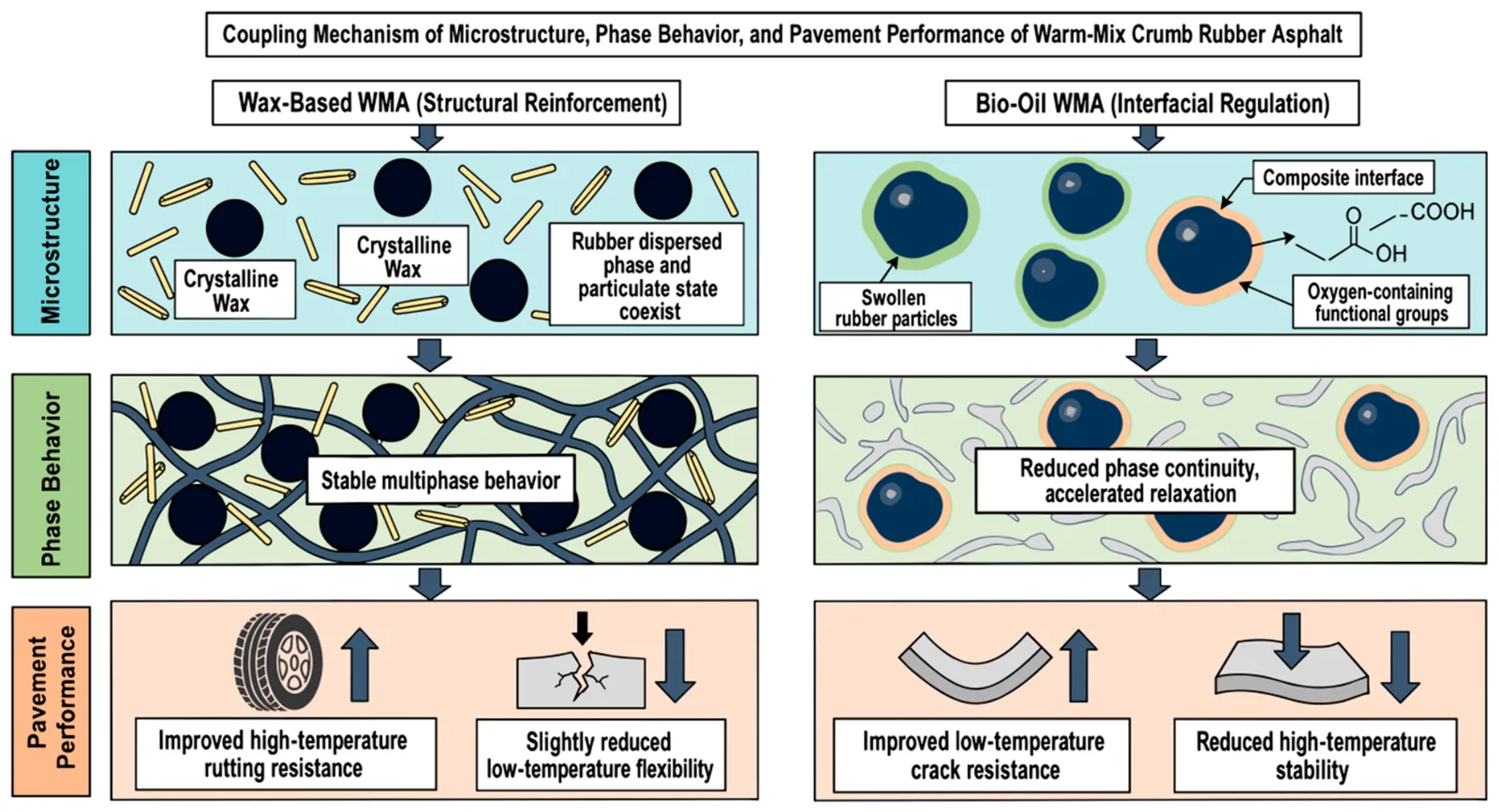
Proposed conceptual interpretation of the relationships among binder composition, rheological phase behavior, and performance-related indicators; the scheme synthesizes the experimental evidence rather than depicting a directly observed microstructure.

Figure 25 summarizes these relationships as a proposed conceptual interpretation rather than a directly imaged microstructure. The scheme integrates the FTIR and rheological evidence into two mechanistic pathways and provides a basis for additive selection. Because binder rheology is a fundamental component of mixture deformation, recovery, and cracking behavior, the observed trends have direct engineering relevance. Mixture-level performance tests, viscosity–temperature and workability measurements, and field validation will further establish service-environment, dosage, and construction-temperature recommendations.

## 5. Conclusions

This study identifies two dosage-dependent binder response pathways rather than a single universally superior warm-mix additive. The conclusions below synthesize the agreement and trade-offs among the FTIR, DSR, MSCR, LAS, and BBR evidence.

(1) The retained principal FTIR bands, together with the coordinated changes in strain dependence and relaxation behavior, support predominantly physical modification. The CRMAL response is consistent with wax-associated physical structuring, whereas the CRMAZ response is consistent with modification of the continuous phase and asphalt–rubber interfacial environment. Agreement among these independent indicators strengthens the proposed interpretation, and direct microstructural characterization can further resolve the corresponding structural evolution.
(2) The wax-based additive shifted the binder toward greater rigidity and elastic recovery. At 3.2 kPa, CRMAL2 combined the lowest *J*_nr_ with the highest *R* among the modified binders, whereas a higher wax dosage was accompanied by a lower LAS-predicted *N_f_* and a less favorable BBR stiffness–relaxation balance. CRMAL2 is therefore the preferred wax dosage among those tested when high-temperature deformation recovery is prioritized, because it avoids the stronger fatigue- and low-temperature-related penalties observed at a 3% dosage.
(3) The bio-oil-based additive shifted the binder toward greater strain tolerance and relaxation, as reflected by a higher *γ*_c_ and tan*δ*, lower Δ*G*′, progressively higher LAS-predicted *N_f_*, and lower *S*(60) with a higher *m*(60). This response is advantageous when fatigue-related damage tolerance and low-temperature relaxation are prioritized. Because *J*_nr_ increased and *R* decreased with a dosage under 3.2 kPa, the selected bio-oil dosage should balance these benefits against the accompanying reduction in high-temperature deformation recovery.
(4) Master-curve, Black-diagram, and Cole–Cole results show that both modified systems have broad, non-ideal relaxation behavior; consequently, the master curves and quasi-terminal slopes are most useful as comparative engineering representations. Additive selection should be multi-objective: the wax-based route favors rigidity and recovery at an appropriate dosage, whereas the bio-oil-based route favors relaxation-related, fatigue-related, and low-temperature indicators. The preferred system therefore depends on climatic conditions and the dominant pavement distress to be controlled.
(5) One-way ANOVA confirmed significant formulation effects on LAS-predicted *N_f_* and on both BBR *S*(60) and *m*(60) (all *p* < 0.001). Tukey’s comparisons showed that all CRMAZ binders had a higher *N_f_* than CRMA at both strain levels and a lower *S*(60) at both BBR temperatures; however, not every numerical difference in *m*(60) or within the wax-based series was significant. These findings strengthen the statistical basis for the fatigue-related and low-temperature binder recommendations while retaining the observed dosage trade-offs.

Statistical comparisons of the replicated LAS and BBR endpoints strengthened the evidence for the fatigue-related and low-temperature binder responses. Future work should extend uncertainty and effect-size reporting across the complete rheological test matrix. Mixture-level rutting, fatigue, and thermal-cracking tests; viscosity–temperature and workability measurements; aging and durability evaluation; and field trials will validate the present binder-level relationships and support quantitative recommendations for construction-temperature reduction, field dosage, and service conditions.

## Figures and Tables

**Table 1:** Binder sample labels and material design.

Specimen Designation	Base Binder	Crumb Rubber Content (wt%)	Warm-Mix Additive Type	Additive Dosage (wt%)	Other Relevant Characteristics
QP	70# base asphalt	0	——	0	Base asphalt, used as the reference for other binder properties
CRMA	70# base asphalt	8	——	0	Wet-process rubber-modified asphalt, used as the reference for warm-mix systems
CRMAL1, CRMAL2, CRMAL3	70# base asphalt	8	Wax-based	1, 2, 3	Wax-based warm-mix rubber-modified asphalt at low, medium, and high dosages
CRMAZ1, CRMAZ2, CRMAZ3	70# base asphalt	8	Bio-oil-based	1, 2, 3	Bio-oil-based warm-mix rubber-modified asphalt at low, medium, and high dosages

**Table 2:** Basic physical indicators of 70# matrix asphalt.

Performance	Result
Penetration at 25 °C (0.1 mm)	68.7
Softening point (°C)	46.9
Viscosity at 135 °C (Pa·s)	0.6
Ductility at 25 °C (cm)	>150
Density at 15 °C (g/cm^3^)	1.036

**Table 3:** Physicochemical indicators of crumb rubber.

Property	Measured Value	Technical Requirement
Moisture	0.48	<1.00
Relative density	1.20	1.10–1.30
Metal content/%	0.01	<0.05
Fiber content/%	0.06	<1.00
Ash content/%	6.20	≤8.00
Carbon black content/%	34.00	≥28.00
Rubber hydrocarbon content/%	58.00	≥42.00
Acetone extract/%	9.00	≤22.00

**Table 4:** Shift factors used for constructing master curves.

Sample	log(*a_T_*) at 5 °C	log(*a_T_*) at 55 °C
QP	3.251888	−1.913882
CRMA	2.898969	−2.018969
CRMAL1	2.898969	−2.148969
CRMAL2	2.898969	−2.098969
CRMAL3	2.898969	−2.220000
CRMAZ1	2.898969	−1.958969
CRMAZ2	2.698969	−1.828969
CRMAZ3	2.598969	−1.798969

**Table 5:** *J*_nr,diff_ and *R*_diff_ values of asphalt samples.

Sample	*J*_nr,diff_ (%)	*R*_diff_ (%)
QP	333	81.6
CRMA	196	65.5
CRMAL1	258.7	69.7
CRMAL2	271.3	65.7
CRMAL3	295.2	73.1
CRMAZ1	207.6	63.1
CRMAZ2	236.9	70.3
CRMAZ3	251.7	74.9

## Data Availability

All datasets generated for this study are available on request from the corresponding author. The raw data cannot be publicly deposited due to confidential university-enterprise cooperation agreements. Please feel free to contact us if you require further information.
